# Decoding the gut microbiota-immune dialogue: from bidirectional axis to therapeutic applications

**DOI:** 10.1186/s12951-026-04376-4

**Published:** 2026-04-14

**Authors:** Yufang Liu, Chao Chen, Huifang Liu, Wei Wang, Xiaoli Zhou, Mengmeng Guo, Juanjuan Zhao, Zhu Zeng, Lin Xu

**Affiliations:** 1 Department of Immunology, Guizhou Medical University, Guiyang, 550025 Guizhou China; 2 Key Laboratory of Cancer Prevention and Treatment of Guizhou Province, Zunyi Medical University, Zunyi, 563000 Guizhou China; 3 Department of Immunology, Zunyi Medical University, Zunyi, 563000 Guizhou China; 4 Collaborative Innovation Center of Tissue, Damage Repair and Regeneration Medicine of Zunyi Medical University, Zunyi, 563000 Guizhou China; 5 Resource Institute for Chinese and Ethnic Materia Medica, Guizhou University of Traditional Chinese Medicine, Guiyang, 550025 Guizhou China

**Keywords:** Gut microbiota, Gut immunity, Bidirectional regulation network, Diseases association, Targeted intervention

## Abstract

The gut microbiota (GM), a highly complex micro-ecosystem residing within the host’s gastrointestinal tract, works in conjunction with the gut immune system to form a precise bidirectional regulatory network, that maintains symbiotic homeostasis and overall host health. Cumulative evidence has demonstrated that the critical impact of the bidirectional causal relationship between the GM and the gut immune system on host development and the dynamic progression of disease. However, many challenges remain in this research field, including the mechanism complexity, therapeutic effect differences due to individual heterogeneity, long-term safety, and clinical transformation bottlenecks) that need to be urgently broken through. Therefore, the in-depth analysis of these issues is of great theoretical and practical significance for clarifying the intrinsic connection between the GM and gut immunity, particularly in elucidating the pathogenesis of related clinical diseases such as inflammatory bowel disease (IBD), tumors, and autoimmune diseases (AD). We systematically outline the interaction mechanisms between the microbiota and the immune system, including compositional structure (microbiota diversity and immune system composition), development and maturation processes (early microbiota colonization and immune system establishment), and functional regulation (immune cell differentiation and maintenance of mucosal barrier integrity), as well as their associations with clinical diseases. Finally, we discuss some key considerations for the developing of innovative treatment strategies, such as microbial-targeted interventions, fecal microbiota transplantation (FMT), and synergistic use of immunomodulatory drugs, with the aim of providing a new paradigm for the precise intervention of related diseases.

**Graphical Abstract:**

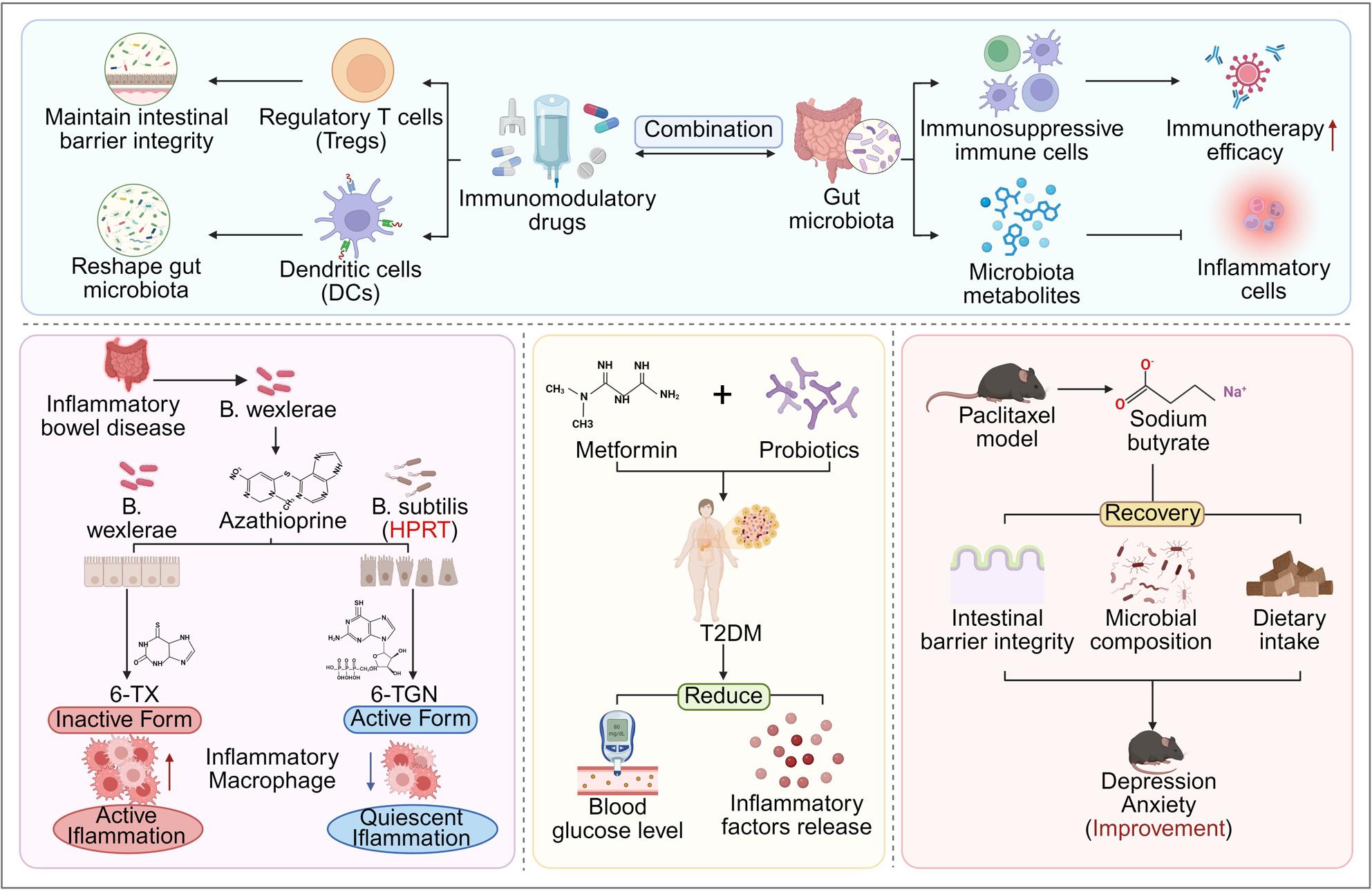

## Highlights

GM-immune axis serves as the core driving force for host development and disease intervention.

Interaction pattern between the GM and the immune system was dynamic across the dimensions of “composition-development-function.”

The vicious cycle of GM and immune imbalance is with heterogeneity in different clinical diseases such as IBD, tumors, and autoimmune disorders.

## Introduction

As the central organ of the human digestive system, the gut performs several vital functions, including digestion, absorption, metabolic detoxification and immune defense [1, 2]. Its structure is divided into two main parts: the small intestine (duodenum, jejunum, and ileum) and the large intestine (cecum, colon, rectum, and anal canal) [3, 4]. It is worth noting that the gut mucosal surface is colonized by a large number of microbial communities of various types. The gut microbiota (GM) constitutes a highly diverse ecosystem, including trillions of bacteria and approximately over 1,000 different microbial species, which play an irreplaceable role in human physiological activities [5, 6]. The community coexists with the host and has functions including regulating body weight and digestive capacity, reducing the risk of viral infection and autoimmune diseases (AD), and affecting the responsiveness of disease treatment drugs [7–9]. Under healthy conditions, beneficial bacteria in the gut are dominant, such as *Bifidobacterium* and *Lactobacillus* [10, 11]. However, GM imbalance can lead to impaired mucosal barrier function, immune homeostasis imbalance, and abnormal response, resulting in pathogen translocation, thus inducing the occurrence of a variety of diseases [12].

The gut is constantly exposed to a microbial environment rich in various pathogens, prompting the body to evolve a highly regionalized gut immune system [6]. This system plays a crucial role in maintaining gut health and systemic immune balance through precise dual regulatory mechanisms: pathogen clearance (such as the secretion of antimicrobial peptides by Paneth cells) and maintenance of symbiotic bacterial tolerance (such as the induction of immune tolerance by regulatory T cells) [13, 14]. It not only serves as a local defense barrier of the intestine to prevent pathogens and toxins from entering the body, but also participates in systemic immune regulation through complex pathways, including cell migration and signal transduction [15]. However, abnormal activation of the gut immune system can induce the occurrence of various diseases, including infectious diseases [16], tumors [17], and autoimmune diseases [18]. Notably, the GM and its metabolites can prevent the occurrence and progrssion of diseases by influencing the function of the gut immune system [19, 20]. However, the specific regulatory mechanisms of the two in diseases are currently unclear and still need to be further analyzed.

## Overview of gut microbiota

The GM is a highly complex and diverse microbial community residing within the host’s gastrointestinal tract. Its composition exhibits considerable inter-individual variation, spatial specificity, and dynamic adaptability. This microbial ecosystem plays a vital role in maintaining host health by modulating key physiological processes such as digestion, metabolism, and immune function through intricate host-microbe interactions [21, 22]. The distribution of GM showes pronounced spatial heterogeneity, primarily located in the stomach, small intestine, and large intestine. Its abundance, diversity, and compositional profile demonstrate a gradient variation along the gastrointestinal axis [23]. This distribution pattern results from long-term co-evolution between the host’s anatomical and physiological characteristics and the microorganisms, influenced by the nutritional supply of different gut segments, the physiological environment (e.g., pH value, oxygen content), and host immune regulation [24–26].

According to the taxonomic framework, the GM can be classified into seven primary hierarchical levels: kingdom, phylum, class, order, family, genus, and species [27]. At the phylum level, the gastrointestinal microbiota in healthy adults is predominantly composed of *Firmicutes* and *Bacteroidetes*, which together account for approximately 90% of the microbial population [28, 29]. Other minor components include *Actinobacteria* and *Proteobacteria*, the relative abundances of these phyla may vary considerably under different physiological or pathological conditions [30, 31]. Based on the influence of the organizational microenvironment and the host-microbe interactions, the GM can be classified into three types: symbiotic bacteria, opportunistic pathogens, and pathogenic bacteria. Importantly, the dynamic balance among these three types is the core mechanism for maintaining gut homeostasis [32, 33]. Taking *Bifidobacterium* as an example, it is a key symbiotic bacterium colonizing in early human neonates [34]. It exerts beneficial effects by inhibiting pathogen growth and regulating the immune system, and its abundance shows a significant age-dependent decline (Infancy is the dominant stage, whereas adulthood constitutes approximately 3 to 6%) [35].

**Fig. 1:**
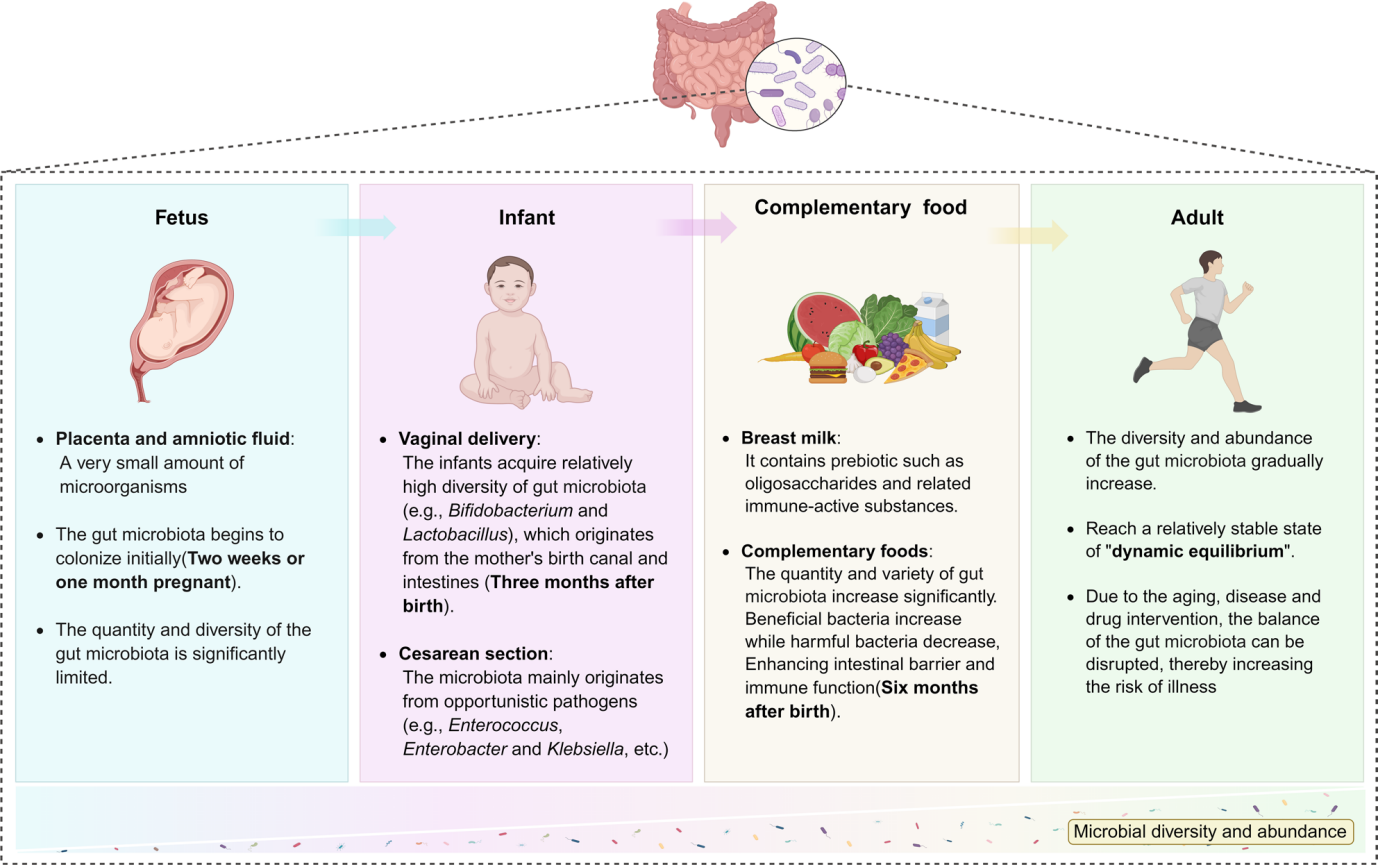
The establishment and dynamic evolution process of the GM in different periods. During the fetal period, minimal levels of microorganisms are detected in the placenta and amniotic fluid, marking the beginning of initial colonization process. The mode of delivery (vaginal delivery versus cesarean section) plays a crucial role in shaping the composition of the infant’s initial microbiota. With the introduction of complementary foods, the diversity and abundance of the microbiota increase significantly, ultimately leading to the establishment of a stable and dynamically balanced microecosystem by adulthood. Created in https://BioRender.com

GM is gradually established from birth, with its density and diversity varying significantly across in different developmental stages of the host (Fig. 1**)** [36, 37]. The early colonizing microbiota serves not only as a core member of the GM but also as a crucial “enlightener” for the development of the neonatal immune system. Through dynamic interactions with the host immune system, it forms an interdependent relationship that promotes the differentiation and development of immune cells and regulates immune responses, significantly contributing to the maintenance of immune tolerance during early life [38, 39]. During this process, prebiotics (such as oligosaccharides) and immune-active substances in breast milk function through a dual mechanism. On one hand, they promote the establishment and rapid proliferation of beneficial bacteria in the infant’s gut [40–42]. on the other hand, they stimulate the differentiation of immune cells, enhance immune tolerance, inhibit excessive inflammatory responses, and promotes the production of secretory IgA (sIgA), thereby forming a mucosal immune barrier that resists pathogen invasion [43, 44]. With the addition of complementary foods in the later stage, the GM of infants gradually increases in both quantity and diversity, approaching the composition of adult microbiota and eventually achieving a relatively stable “dynamic balance” state [45, 46].

However, this balance can be disrupted by factors such as aging, diseases, and drug interventions (especially antibiotics), all of which can disturb the homeostasis of the microbiota [37]. This disruption leads to GM imbalance accompanied by immune dysfunction, including impaired immune tolerance, reduced defense capability, or chronic inflammation. These changes in the microbiota structure indicate that abnormal signal output can disrupt normal immune response patterns.

## Overview of gut immune system

**Fig. 2:**
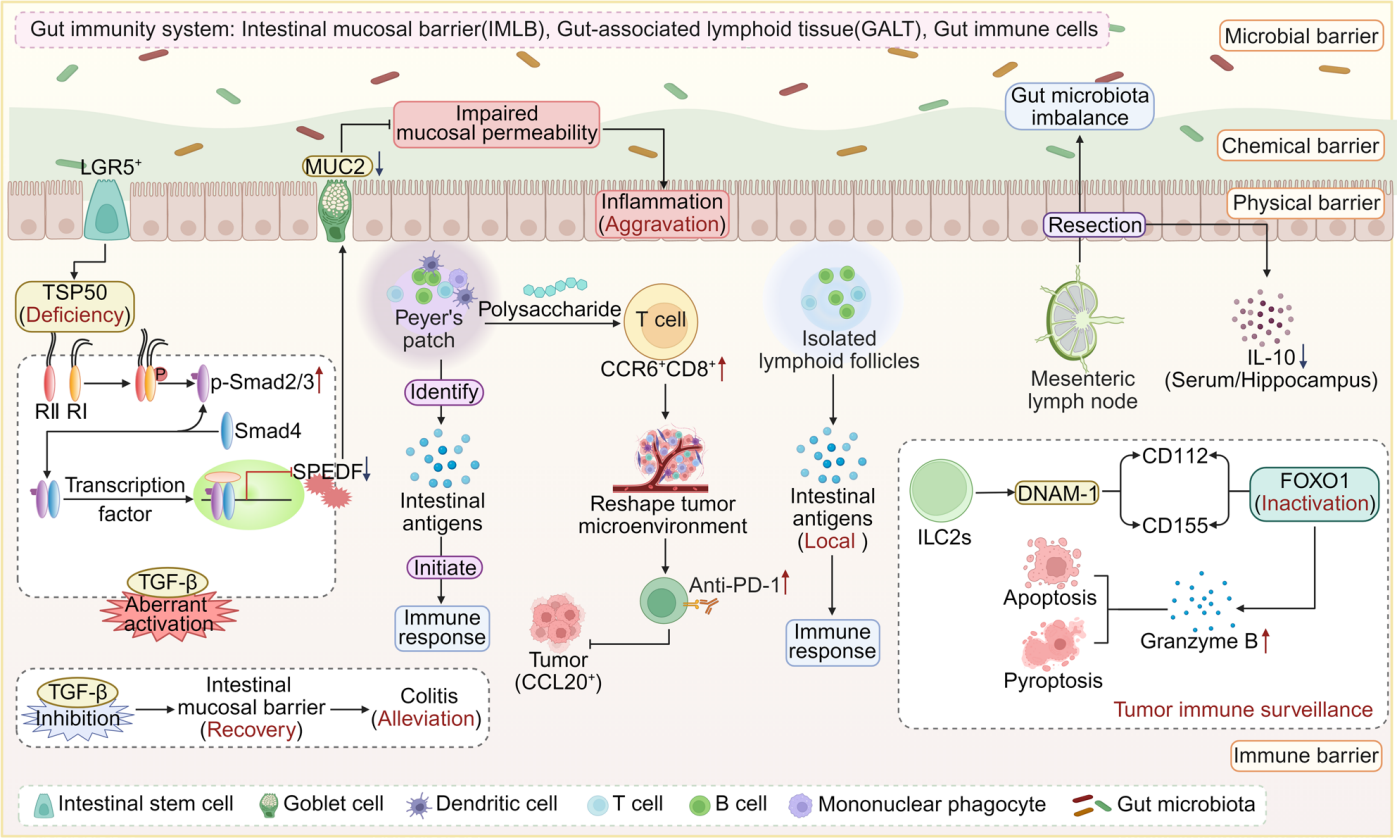
The multi-layered defense, homeostasis maintenance, and immune surveillance mechanisms of the gut immune system. ,Serving as the central mucosal immune barrier, the gut immune system resists pathogenic invasion and maintains symbiotic tolerance through the gut mucosal barrier, which comprises microbial, chemical, physical, and immune barrier components. Compromise of this barrier’s integrity can lead to diseases such as IBD and tumors. A deficiency of TSP50 in the ISCs indirectly activates the TGF-β signaling pathway, thereby inhibiting goblet cell differentiation and resulting in impaired mucosal permeability. Targeted inhibition of the TGF-β signaling pathway can reverse this damage. The GALT, including PP, ILF, and MLN, plays a pivotal role in antigen sampling and immune induction, orchestrating the progression from an initial response to a localized reaction and ultimately systemic integration. Locally distributed immune cells collaborate to maintain mucosal homeostasis. Notably, specific cell types such as ILC2s induce the inactivation of the negative regulatory factor FOXO1 via the DNAM-1-CD112/CD155 pathway, directly triggering both pyroptosis and apoptosis in tumor cells, thereby facilitating immune surveillance. Created in https://BioRender.com

The gut immune system constitutes a sophisticated defense network against pathogens, comprising the intestinal mucosal barrier (IMLB), gut immune cells, and gut-associated lymphoid tissues (GALT) [47–49] **(**Fig. 2**)**. As the primary line of defense, the IMLB is consists of coordinated interactions among microbial, chemical, physical, and immune components. Maintaining its structural and functional integrity is essential for sustaining homeostasis [50]. Studies have shown that TSP50 can maintain the integrity of the IMLB by regulating the TGF-β signaling pathway and alleviating DSS-induced colitis [51]. However, impaired barrier function weakens the ability to clear pathogens, leading to gut homeostasis imbalance, and abnormal immune activation, and ultimately inducing diseases such as inflammatory bowel disease (IBD) and irritable bowel syndrome (IBS) [52–54]. Gut immune cells exhibit a region-specific distribution pattern. The gut epithelium predominantly contains tissue-resident T cells, whereas the lamina propria harbors a diverse population of both innate and adaptive immune cells [55, 56]. These immune cells play a critical role in maintaining immunological homeostasis and preserving mucosal barrier integrity through the activation of coordinated immune responses, thereby preventing the development of gut pathologies [57]. For example, human type 2 innate lymphoid cells (ILC2s) actively contribute to tumor immune surveillance by inducing tumor cell death via a dual mechanism involving both pyroptosis and apoptosis [58] **(**Fig. 2**)**.

GALT is the largest immune organ in the human body and serves as the induction site for key antigen sampling and adaptive immunity within the gut wall, which is crucial for maintaining gut homeostasis [59, 60]. Its structure primarily includes Peyer’s patches (PP), isolated lymphoid follicles (ILF), and mesenteric lymph nodes (MLN) [60]. These three components collaborate to establish a multi-layered immune defense network. PP, located in the distal ileum, is rich in various immune cells and is responsible for antigen recognition and immune initiation [61, 62]. For example, Lactobacillus-derived exopolysaccharides can increase the number of CCR6^+^CD8^+^ T cells in the PP, remodeling the tumor microenvironment to enhance immunotherapy [63]. ILF, distributed in the gut mucosa, primarily mediates local antigen responses through B cells [64, 65]. Finally, MLN integrates antigens and immune cells via the lymphatic network and coordinates both local and systemic immunity. The absence of MLN may lead to dysbiosis and decreased IL-10 level, which can affect the neurobehavior of mice [66–68] **(**Fig. 2**)**.

The initial development of the human gut immune system begins during the fetal period. However, its overall immune response remains immature [69]. Its development and maturation constitute a complex, dynamic process shaped by the host’s genetic program and the microbial environment [70–72]. After birth, the colonization of GM becomes a key exogenous stimulus driving the development and maturation of gut immune cells [73, 74]. Breastfeeding affects the colonization pattern of early GM through passive immunity factors such as immunoglobulins and lactoferrin [75, 76]. Beyond early colonization, once a mature GM is established, exogenous interventions (such as the uptake of probiotic fermented milk) can increase the number of gut macrophages (Mø) and dendritic cells (DCs), as well as upregulate the expression of pattern recognition receptors TLR-2 and the mannose receptor CD206 [77, 78]. Additionally, later dietary patterns, such as the ketogenic diet (KD), can significantly decrease in the number of Th17 cells in the gut by reducing the relative abundance of beneficial bacteria in the gut of mice, thereby disrupting the mucosal immune balance [79]. Collectively, these findings reveal the dynamic plasticity of gut immune system function throughout the life cycle.

## Interaction mechanism between gut microbiota and gut immunity

### Gut microbiota regulates gut immunity

Recent studies have revealed that the GM not only participates in nutrient metabolism but also dynamically regulates the balance of the mechanism of gut immune response through molecular interactions between the microbiota and the host [12, 80]. This microbe-mediated immune regulation both initiates effective defenses against pathogen invasion and prevents overreactions to symbiotic microbiota and food antigens [81]. However, when this microecological balance is disrupted, it may trigger a cascade of events ranging from gut inflammation to systemic immune diseases [82, 83], making the analysis of the molecular pathways involved in microbial regulation of immune homeostasis become a current frontier and hot topic. Notably, during early colonization, maternal microorganisms can regulate the development of the offspring’s immune system through vertical transmission. GM colonization in pregnant female mice can significantly increases the number of ILC3s and F4/80^+^CD11c^+^ monocytes in the gut of their young mice [84]. Studies in germ-free (GF) mice further confirm that the absence of GM results in a significant decrease in the number of invariant natural killer T cells (iNKTs) in mesenteric lymphoid tissue (ileum), liver, spleen, and thymus. This difference is detectable after weaning, indicating early initiation and persistence of of the immune system regulation by GM [85].

The GM plays a critical role in the development, maturation, and function of the immune system by recognizing microbial molecules through pattern recognition receptors (PRRs), regulating immune cell differentiation, and producing metabolites such as short-chain fatty acids (SCFAs) [86–89]. Moreover, the composition of the GM also influences immune system development and modulates immune mediators, thereby affecting gut barrier function [90]. Existing studies have shown that the gut mucosal immunity of germ-free animals is underdeveloped, the MLNs are smaller, and the number of immune cells is reduced. This includes plasma cells that produce IgA, the number of intraepithelial αβT cell receptor CD8^+^ T cells and CD4^+^LPT cells are reduced, resulting in a diminished ability of the host to resist pathogens [91, 92]. These finding suggest that early microbiome colonization is essential for optimal immune system development. SCFAs, the primarily products of fermentation by fibrous bacteria in the colon, including acetate (C2), propionate (C3), and butyrate (C4), can cross the gut epithelium and interact with host cells, thereby influencing immune responses and diseases risk [93, 94]. For example, butyrate in SCFAs regulates Th1 and Th17 cells differentiation by inhibiting histone deacetylase and controls the development of T cells induced colitis by upregulating the expression of B-lymphocyte-induced maturation protein 1 (Blimp1) and promoting IL-10 production [95]. Additionally, the GM significantly regulates the immune microenvironment of the MLN. For instance, studies have confirmed that GM intake effectively reduces the proportion of Th1 cells (CD4^+^IFN-γ^+^) in the MLN [96], while increaseing the proportion of Treg cells (CD25^+^Foxp3^+^). Consequently, immune homeostasis in the liver and gut under HF diet pattern is maintained, and systemic metabolic disorders are effectively improved [96].

The metabolites produced by the microbiota can also activate immune cells in the gut and participate in regulating gut immunity to maintain host health [97, 98]. *Lactobacillus*, as the main genus of GM, has many probiotic functions [11, 99]. For example, studies have show that *Lactobacillus rhamnosus* GG (LGG) can influence the proportions of CD3^+^ T lymphocytes in the spleens (SPLs), MLN, PPs and lamina propria lymphocytes (LPLs), including total CD3^+^T cells, CD3^+^CD4^+^T cells and CD3^+^CD8^+^T cells [99]. Furthermore, LGG effectively increases the expression of Th1-type cytokines (IFN-γ) and Th2 cytokines (IL-4) in CD4^+^T cells [99]. Furthermore, the GM can enhance Treg cell differentiation by activating the aryl hydrocarbon receptor (AhR), thereby promoting the differentiation of naïve CD4^+^T cells to differentiate into immunosuppressive FoxP3^+^ Treg cells rather than pro-inflammatory Th17 cells [100, 101]. As antigen-presenting cells, DCs can secrete cytokines and chemokines to induce and maintain immune tolerance [102, 103]. Campbell et al. demonstrated that the secondary bile acid isodeoxycholic acid (isoDCA) induces Foxp3 expression by modulating farnesoid X receptor (FXR) signaling [101], thereby reducing the immune-stimulatory function of DCs. Moreover, the bacterial community producing isoDCA relies on CNS1 to increase the proportion of RORγt^+^ Treg cells in the colon without affecting Th17 cells [101]. Notably, a specific subset of antigen-presenting cells in the gut-tolerogenic dendritic cells (tolerogenic DCs), plays a central role in maintaining peripheral tolerance and characterized by the co-expression of CD103 and CD11b [104]. On one hand, they secrete retinoic acid and TGF-β, inducing the differentiation of naive T cells into Foxp3^+^ Tregs [105, 106], while inhibiting the differentiation of pro-inflammatory Th17 cells [107], thereby establishing immune tolerance to the commensal microbiota. On the other hand, they also participate in regulating the homeostasis of intestinal epithelial cells, promoting the effector functions and antibacterial defense of local T cells, and jointly maintaining the integrity of the gut barrier [104]. For example, Binf can promote the accumulation of CD103^+^ tolerogenic DCs in the GALT and alter the composition of the GM, especially by increasing the proportion of Dorea and decreasing the proportion of Ralstonia, ultimately alleviating the allergic reaction caused by tropomyosin (Tm) protein [108]. These findings collectively suggest that the GM can trigger gut immunity by promoting the development of immune organs and regulating immune cell functions, thereby maintaining gut immune homeostasis and enhancing overall immune functionality.

### Regulation of gut microbiota by gut immunity

The gut immune system serves not only as a defensive barrier for the host but also an “ecological manager” that regulates the homeostasis of the microbiota [109, 110]. It accurately identifies between symbiotic bacteria and pathogens by secreting immunoglobulin A (IgA) and regulating the expression of antimicrobial peptide, playing a central role in maintaining bacterial diversity, spatial distribution, and metabolic activity [111, 112]. This immune-mediated “bidirectional regulation” mechanism not only ensures the ecological balance of the microbiota, but also prevents excessive inflammatory responses through immune tolerance. However, disruption of gut immune function can lead to various pathologies, including persistent infections, autoimmune disordes, and even malignant [113, 114]. Therefore, understanding the molecular interaction mechanisms between gut immunity and the GM is particularly important for the targeted regulation of microecological balance.

Gut epithelial cells protect the gut mucosa from both symbiotic and invasive pathogenic microorganisms by forming a multi-dimensional barrier system, which includes physical and chemical barriers [115]. The mucus layer secreted by goblet cells serves as the primary defense interface, maintaining the balance of GM colonization and growth by providing nutrients such as mucin. Mucin produced by goblet cells forms the mucus layer, effectively isolating GM from epithelial cells and protecting gut epithelial cells from pathogen invasion and physical damage [116–118]. Additionally, the gut epithelial cell-derived protein Lypd8, as a member of the Ly6/PLAUR superfamily, inhibits the chemotactic movement of Gram-negative flagellated bacteria by specifically binding to flagellate bacteria and restricting their penetration into the colon mucus layer, thereby preventing invasion of colon epithelial cells and maintaining gut homeostasis [119]. This suggestes that the physical barrier function of the mucus layer is essential for protecting the host’s gut health. Notably, defects in the mucus layer under pathological conditions can trigger inflammatory cascade reactions. Studies have shown that oral administration of thiol-modified hyaluronic acid (HASH) hydrogel precursor solution can form an artificial mucus coating in inflamed gut regions, preventing microbial invasion and alleviating abnormally activated immune response [120]. At the same time, the HASH intervention also significantly increased GM diversity and enhanced the abundance of SCFAs associated bacteria [120]. These studies suggest that a multidimensional synergistic mechanisms-comprising barrier repair, immune regulation, and microbiome remodeling-may provide novel therapeutic strategies for gut-related diseases.

Ribonuclease 4 (RNase4), as an epithelial-derived antimicrobial peptide, is highly expressed in Paneth cells and goblet cells [121]. Studies have demonstrated that RNase4 can regulate the composition of the GM by targeting lytic *Parasutterella*, thereby upregulating the expression of indoleamino-2,3-dioxygenase 1 (IDO1) in the gut epithelial cells. This promotes the biosynthesis of its metabolites, kynurenic acid and xanthurenic acid, and ultimately reducing the susceptibility to experimental colitis [121]. In addition to innate immune proteins, adaptive immune components such as secretory immunoglobulin A (SIgA) drive the microbiota transition from a neonatal state (dominated by *γ-Proteobacteria*, especially *Enterobacteriaceae*) to a mature state (dominated by *Bacteroidetes* and *Firmicutes*) [122]. Notably, significant GM dysregulation has been observed in IgA-deficience mouse models, including markedly reduced overall microbial diversity, substantial changes in bacterial composition, increased susceptibility to gut inflammation, and enhanced bacterial translocation [112, 123, 124]. Furthermore, existing studies have confirmed that impaired IMLB integrity and inflammatory responses are closely associated with abnormal accumulation of bacterial flagellin [125, 126]. For example, despite elevated total IgA levels in the gut of TLR5-deficient (TLR5^−/−^) mouse models, flagellin-specific IgA is severely lacking, allowing *Proteobacteria* and *Firmicutes* to penetrate the small intestinal villi and thus breach the colonic mucosal barrier [126]. It was further found that in vitro, flagellin-specific IgA inhibited bacterial motility while downregulating the expression level of the flagellar gene (FliC) in *E. coli* [126]. These studies reveal a multilayered microbiota regulatory network, spanning from innate immune proteins to adaptive immunoglobulins, whose core mechanism primarily depends on the specific recognition and functional modulation of immune molecules target microbiota antigens.

Hydroxychloroquine (HCQ), as a potential antiproteinuric agent in IgA nephropathy (IgAN), has been shown to significantly mitigate pathological changes associated with IgA deposition, mesangial matrix proliferation, and increased glomerular inflammatory cell infiltration in IgAN rat models [127]. Furthermore, HCQ can impaired IMLB function caused by IgAN, thereby reducing gut permeability and microbial dysbiosis [127]. In addition to pharmacological interventions, the regulatory B cell (Breg) subpopulation regulates GM homeostasis by secreting IL-10 and antigen-specific IgG, and influencing the diversity of early microbial communities through the regulation of energy metabolism [128, 129]. Human umbilical cord mesenchymal stem cells (HUMSCs) have demonstrated potential in alleviating DSS-induced colitis by modulating the Treg-IgA response, promoting IgA secretion, and facilitating the recovery of GM [130]. Additionally, RegIIIγ (Reg3g), a key effector of the mucosal physical barrier, is a secreted C-type lectin that establishes a “sterile zone” approximately 50 μm wide on the surface of the small intestine mucosa, effectively confining the microbiota to the gut lumen [131, 132]. Conversely, in RegIIIγ-deficient mice, the loss of separation between host tissues and bacteria leads to increased bacterial colonization on the epithelial surface of the small intestine and heightened abnormal activation of the gut adaptive immune response by the microbiota [132], underscoring the critical role of RegIIIγ-mediated spatial restriction of the microbiota in maintaining immune homeostasis.

The gut immune system maintains microbiota homeostasis through a multi-level regulatory network. Gut immunoglobulins, as a key effector molecules of the mucosal barrier, precisely regulate bacterial colonization by targeting and binding to bacterial surface antigen, thereby achieving a dynamic balance between symbiotic bacteria selection and pathogen clearance. Meanwhile, the antimicrobial peptides released by innate immune cells provide broad-spectrum chemical defense. This multi-level immune regulatory network not only ensures the stability of the microbiota’s ecological diversity but also enables the adaptive regulation of the gut microenvironment through immune-microbiota interactions, providing a molecular framework for analyzing the immune-mediated microecological balance.

## Diseases caused by gut microbiota and gut immune imbalance

### Inflammatory bowel diseases

IBD is a chronic, progressive immune-mediated idiopathic gastrointestinal inflammatory condition that affects approximately 1% of the global population [133, 134]. It primarily includes two types: Crohn’s diseases (CD) and Ulcerative colitis (UC). The main clinical features of IBD include chronic diarrhea, with or without bleeding, abdominal pain, and weight loss [133, 135, 136]. The pathogenesis of IBD is complex and involves the interaction of multiple factors, such as environmental factors, genetic predisposition, GM imbalance and mucosal immune system dysfunction [137–139]. Notably, dysregulation of the GM plays a key role in the progression of IBD. Numerous studies have demonstrated that the composition and diversity of the GM in IBD patients differ significantly different from those in healthy individuals, particularly with a reduction in beneficial bacteria, which may directly exacerbate the occurrence and progression of the disease [140, 141]. Therefore, an in-depth analysis of the regulatory mechanisms of the GM in IBD can provide a crucial scientific basis for the subsequent development of novel therapeutic strategies.

Further studies have shown that IBD primarily results from an imbalance in the interaction imbalance between the host and the GM, which is mainly reflected in three aspects: GM disturbance, abnormal immune response, and impairment of IMLB [142]. Specifically, disruption of GM homeostasis can lead to excessive colonization and invasion of pathogenic bacteria in the gut, thereby trigger abnormal host immune responses and accelerating the progression of IBD [137]. For example, in a two-sample Mendelian randomization analysis found that the abundances of *Coprococcus 2*, *Oxalobacter*, and *Ruminococcaceae* were found to be positively associated with IBD risk [143]. The clinical control study further showed that *Shigella dysenteriae*, *Erysipelotrichaceae bacterium*, *E. coli* and *Escherichia marmotae* were more abundant in the CD group, whereas *vallismortis*, *Lactobacillus ruminis*, *Alicycliphilus dentirficans* and *Lachnospira eligens* were more abundant in the UC group [144]. These findings suggest that structural changes in the GM may be a core factor driving the development of IBD.

In individuals with a familial genetic and environmental predisposition, impaired gut immune regulation can trigger chronic immune overactivation, leading to an increase in gastrointestinal lesions such as IBD [145]. Studies have found that butyrate can inhibit the release of pro-inflammatory cytokines (e.g., IL-6, TNF-α, IFN-γ), chemokines (e.g., CCL3, CCL4, CXCL1, and IL-8), and calprotecin (S100A8 and S100A9) in Mø and neutrophils. At the same time, there was no significant effect was observed in healthy individuals [146, 147]. Animal studies further demonstrated that oral butyrate inhibited neutrophil-related immune responses-such as proinflammatory mediators and neutrophil extracellular trap (NET) formation-blocked NF-κB signaling, and reversed histone H3 acetylation, thereby significantly improving mucosal inflammation in DSS induced colitis in mice [146, 147]. These findings suggest that the GM may be involved in regulating the occurrence and progression of IBD by modulating immune cell functions.

In addition to the influence of metabolites, the abnormal function of immune cells themselves is also an important cause of IBD. For example, recent studies have found that Mø not only control gut infections and maintain gut homeostasis by recognizing pathogens but also prevent chronic inflammation through negative feedback mechanisms [148, 149]. T cell immunoglobulin and mucin domain 3 (Tim-3), as a multifunctional immunomodulator, can inhibit the onset of gut inflammation [150–152]. For instance, studies have shown that the knockout of Tim-3 in Mø leads to the recruitment of neutrophils and induces their necrotic apoptosis, resulting in damage to the IMLB and triggering a vicious cycle of colitis development [153]. Furthermore, the injection of Mesenchymal Stromal Cells (MSCs) has demonstrated a significant reduction in gut inflammation in both animal and human studies. Research indicates that human intestinal MSCs (iMSCs) can markedly reduce the infiltration of immune cells into the colon and restore the recruitment and differentiation of certain gut monocytes [142]. More importantly, iMSCs significantly improved the inflammatory response in mouse models of colon-associated colon cancer (CAC) by restoring microbiota balance, such as increasing the abundance of *Akkermansia* and decreasing inflammation-associated bacteria genera, including *Alistipes* and *Turicibacter*) [142].

The stability of the GM depends on the precise regulation of the host immune system, in which antimicrobial peptides (AMPs) and IgA play crucial roles. Studies have shown that dysfunction of these two immune effector molecules can directly alter the composition of the microbiota, thereby exacerbating intestinal inflammation. For example, in IBD, the citrullination of cathelicidin (CAMP) mediated by peptidyl arginine deiminase 4 (PAD4) significantly reduces CAMP protein levels, weakening its regulatory effcet on the GM [154]. Macroproteomic analysis further confirmed that the absence of CAMP directly led to changes in the composition of 15 bacterial families in the gut, indicating that a decrease in AMP expression or its inactivation due to post-translational modification can disrupt GM stability, thereby promoting the occurrence and progression of intestinal inflammation [154].

Furthermore, abnormal quality and quantity of IgA responses are key factors driving the imbalance of the microbiota. On one hand, the widespread impairment of IgA production alters the composition of the microbiota. For example, an increase in the genus *Alcaligenes*, which produces alkaline substances, is observed in Peyer’s patches, and segmented filamentous bacteria are enriched on intestinal epithelial cells [155]. On the other hand, the specific abnormalities in IgA subclasses can also alter the reactivity to particular bacterial communities. For instance, in patients with IBD, the IgA1 reactivity to the opportunistic pathogen Escherichia coli is enhanced, while the IgA reactivity to the beneficial bacterium *Akkermansia muciniphila* is reduced. This further demonstrates that the abnormalities in both the quality and quantity of IgA secretion can lead to specific changes in the composition of the GM [156]. The disruption of IgA function, such as its enrichment effect on potential pathogenic bacteria like the *Proteobacteria*, is a typical feature of microbiota imbalance in inflammatory conditions such as colitis [157]. In summary, the inactivation of antimicrobial peptides and the dysfunction of IgA collectively constitute an important molecular basis for the “immune imbalance-microbiota imbalance” vicious cycle in IBD.

### Colorectal cancer

**Fig. 3:**
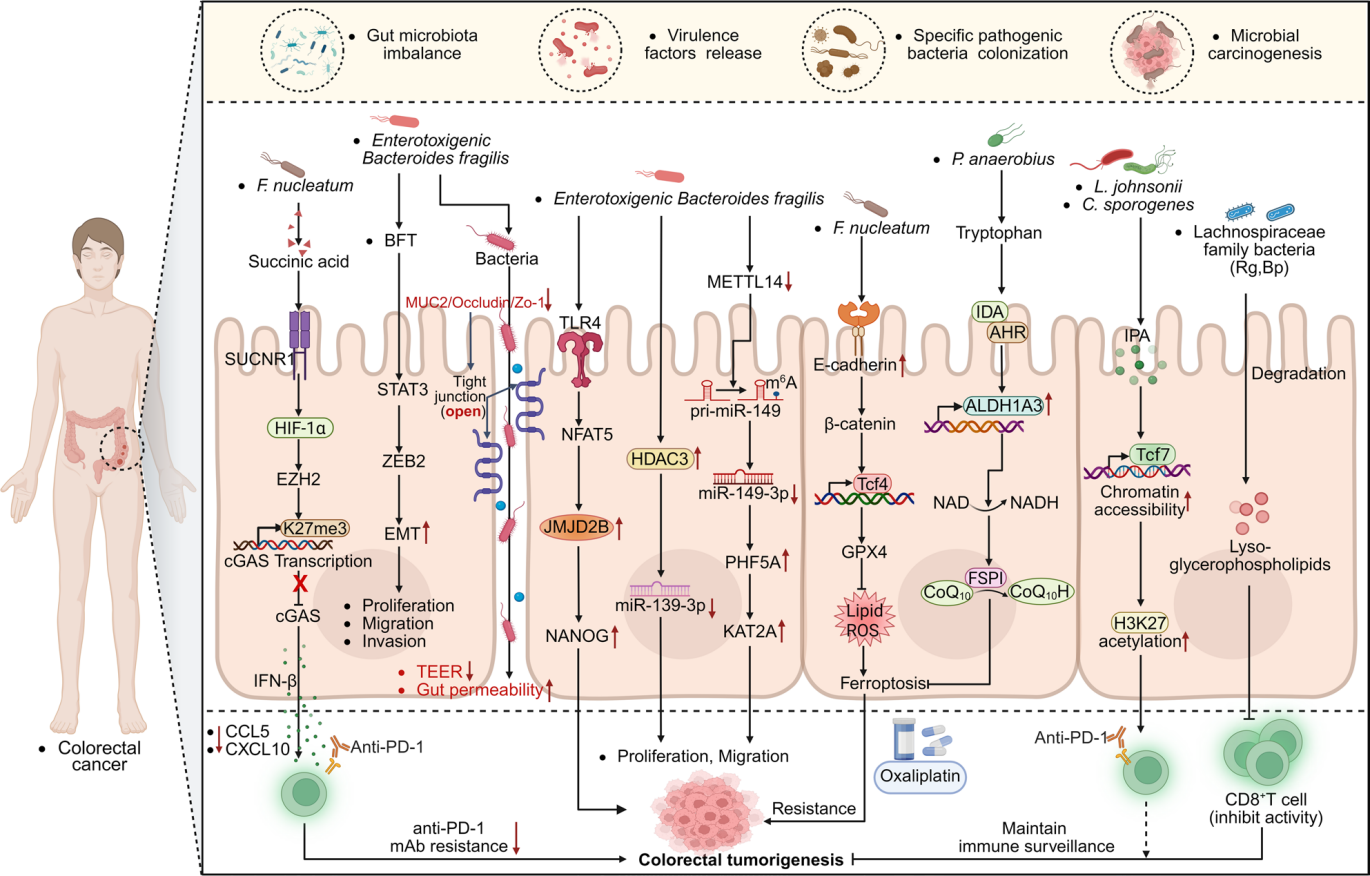
The interaction mechanism between GM and CRC and the regulatory role of immunotherapy. The occurrence and progression of CRC are influenced by a multitude of factors, including GM imbalance, colonization by specific pathogenic bacteria, the release of virulence factors, and microbe-related carcinogenic mechanisms. The GM contribute to CRC development through dual mechanisms that both promote and inhibit cancer. Pathogenic bacteria, such as *F. nucleatum*, ETBF and *Streptococcus* species, compromise the gut barrier by secreting toxins like BFT. These actions activate signaling pathways, including STAT3/ZEB2 to promote EMT, inhibit the cGAS-IFN-β pathway, and drive metabolic reprogramming via metabolites such as succinic acid and indole derivatives (IDAs). Consequently, this weakens CD8^+^ T cell functionality and induces therapeutic resistance. Conversely, beneficial bacteria, such as *L. johnsonii* and *C. sporogenes*, enhance CD8^+^ T cells activity through tryptophan metabolism, producing IPA, thereby improving the efficacy of ICB therapy. Additionally, tissue-resident bacteria, such as Rg and Bp, maintain the immune surveillance capabilities of CD8^+^ T cells by degrading Lyso-glycerophospholipids. Created in https://BioRender.com

Colorectal cancer (CRC) is a common malignant tumor of the digestive tract with high morbidity and mortality [158]. The GM primarily colonizing in the colorectal region, plays a crucial role in the occurrence and progression of CRC by releasing metabolites, proteins, and macromolecules, as well as interacting with host colon epithelial cells and immune cells [159]. Studies have shown that disturbances in the GM [160], colonization by specific pathogens [161, 162], release of virulence factors [163], and microbial-related carcinogenic mechanisms can directly or indirectly promote CRC progression **(**Fig. 3**)**

Metagenomic analysis has further revealed the characteristic changes in CRC-associated bacterial groups. Compared with healthy individuals, the abundance of cancer-promoting bacteria such as *Bacteroides*, *Fusobacterium*, and *Peptostreptococcus* is significantly increased in the GM of CRC patients [164]. For example, *Enterotoxigenic Bacteroides fragilis* (ETBF) has been shown to promote CRC development through a dual mechanism. On the one hand, ETBF disrupts the tight junctions and villus structure of the gut barrier by secreting Bactero fragilis toxin (BFT), resulting in increased gut permeability and decreased transepithelial electrical resistance (TEER) [165]. On the other hand, ETBF promotes the proliferation, migration, and invasion of CRC cells by downregulating the expression of tumor-suppressive miRNAs (such as miR-149-3p, MiR-139-3p) and upregulating the expression of the histone demethylase JMJD2B) [166–168]. Animal experiments have confirmed that ETBF colonization can significantly increases tumor burden and IMLB damage in AOM/DSS-induced CRC models, and the mechanism is closely related to BFT’s ability to accelerate epithelial-mesenchymal transformation (EMT) and tumor invasion through activation of the STAT3/ZEB2 signaling axis [165, 167] **(**Fig. 3**)**. These findings not only clarify the carcinogenic role of specific microbiome of the microbiome, but also provide a basis for therapeutic strategies targeting microbiome-host interactions in CRC.

*Fusobacterium nucleatum* (*F. nucleatum*) is a gram-negative, obligate anaerobic bacterium belonging to the genus Fusobacterium. It commonly exists as a symbiotic bacteria in humans and animals [169, 170]. Studies have shown that *F. nucleatum* can activate the succinic acid receptor (SUCNR1)-HIF-1α-EZH2 signaling axis in tumor cells by secreting the metabolite succinic acid,. This activation inhibits the cGAS-IFN-β pathway and reduces the secretion of Th1 chemokines CCL5 and CXCL10 [171], thereby weakening the chemotactic and activation function of CD8^+^ T cells and ultimately diminshing the efficacy of PD-1 monoclonal antibody therapy against CRC [171]. Additionally, *F. nucleatum* is associated with immunoresistance in CRC. For example, *F. nucleatum* can induce chemotherapy resistance of CRC to oxaliplatin by inhibiting ferroptosis through upregulation of the E-cadherin/β-catenin/GPX4 signaling axis [172]. Beyond *Clostridium*, other GM may also influence CRC progression through metabolites. Recent studies have identified that trans-3-indoleacrylic acid (IDA), produced through tryptophan metabolism by *Peptostreptococcus*, as an endogenous ligand of the arylhydrocarbon receptor (AHR), IDA activates AHR signaling [173], transcriptionally upregulates the expression of aldehyde dehydrogenase 1 family member A3 (ALDH1A3), drives NADH metabolic reprogramming, and promotes the development of CRC, the absence of AHR or ALDH1A3 largely abolishes the cancer-promoting effects of IDA [173] **(**Fig. 3**)**. These findings highlight the critical role of GM and their metabolites in the initiation, progression, and treatment resistance of CRC.

In recent years, some specific strains in the gut have been found to significantly enhance the anti-tumor effects of ICB therapy by modulating both the innate and adaptive immune responses [174–176]. For example, the abundance of the symbiotic bacterium “*Lactobacillus johnsonii*” positively correlates with responsiveness to ICB therapy. Supplementation with *Lactobacillus Johnson* or its metabolite indole-3-propionic acid (IPA) significantly enhances the efficacy of CD8^+^ T cell-mediated immunotherapy with αPD-1 [177]. Mechanistic studies have shown that IPA, produced synergically by *Lactobacillus johnsonii* and *Clostridium sporogenes* promotes the expansion of CD8^+^ T cells prior to stem-like depletion by increasing the H3K27 acetylation levels in the Tcf7 super-enhancer region, thereby improving the response of CRC to ICB [177]. In addition to directly regulating the treatment response, the GM also participates in the process of tumor immune surveillance, influencing initiation and progression [178, 179]. Analysis of normal (N), para-cancerous (P), and cancerous (C) colorectal tissues from different CRC patients reveaaled that tissue-resident bacteria *Ruminococcus gnavus* (Rg) and *Blautia producta* (Bp) can degrade lyso-glycerophospholipids, inhibit the activity of CD8^+^ T cells, and maintain their immune surveillance, thereby effectively controlling the progression of CRC [180] **(**Fig. 3**)**.

Recent studies have identified some new mechanisms. These mechanisms include the translocation of specific bacteria and their direct inhibition of local immune cells, as well as the collapse of the innate defense barrier caused by the loss of core AMP functions. Research has shown that during colitis, the lymphoid tissue-resident commensal bacterium Alcaligenes faecalis (A. faecalis) can translocate from Peyer’s patches to the colon tissue [181]. This process not only weakens the immune response capabilities of B cells, T cells, DCs in Peyer’s patches bur also disropts the homing of IgA^+^ B cells and compromises the integrity of the gut barrier by increasing the acetylation of adherens junction proteins. Ultimately, these changes promote the progression of inflammation-assocated CRC [181]. This finding underscores the crucial role of the IgA system in restricting the translocation of specific bacteria and preventing the progression of CRC.

Meanwhile, through single-cell transcriptome analysis of tumor tissues from 42 CRC patients, it was found that the tumor-enriched bacterium Fusobacterium nucleatum (Fn) could disrupt the interaction network between tumor-associated macrophages (TAMs) and IgA plasma cells, thereby inhibiting the development of IgA plasma cells and the production of secretory IgA (sIgA) [182]. The reduction of sIgA leads to an increased in bacterial load within the tumor, which in turn triggers persistent chronic inflammation, creating a positive feedback loop of “increased bacterial load-immune suppression-bacterial proliferation”. This “IgA maturation disorder” module is significantly associated with poorer prognosis in patients [182]. In addition to immunity dysfunction, weakening of innate immune defense is also acritical factor contributing to the occurrence of CRC. AMP serve as the first line of defense to maintain GM balance, and their functional deficiency directly leads to dysbiosis [183]. For example, the antimicrobial peptide LL-37/CRAMP maintains mucosal homeostasis by preventing bacteria colonization on the epithelium [184]. Loss of CRAMP results in dysbiosis and a pro-inflammatory state, which together significantly increase the host’s susceptibility to colon tumor develpment [185].

### Autoimmune diseases

**Fig. 4:**
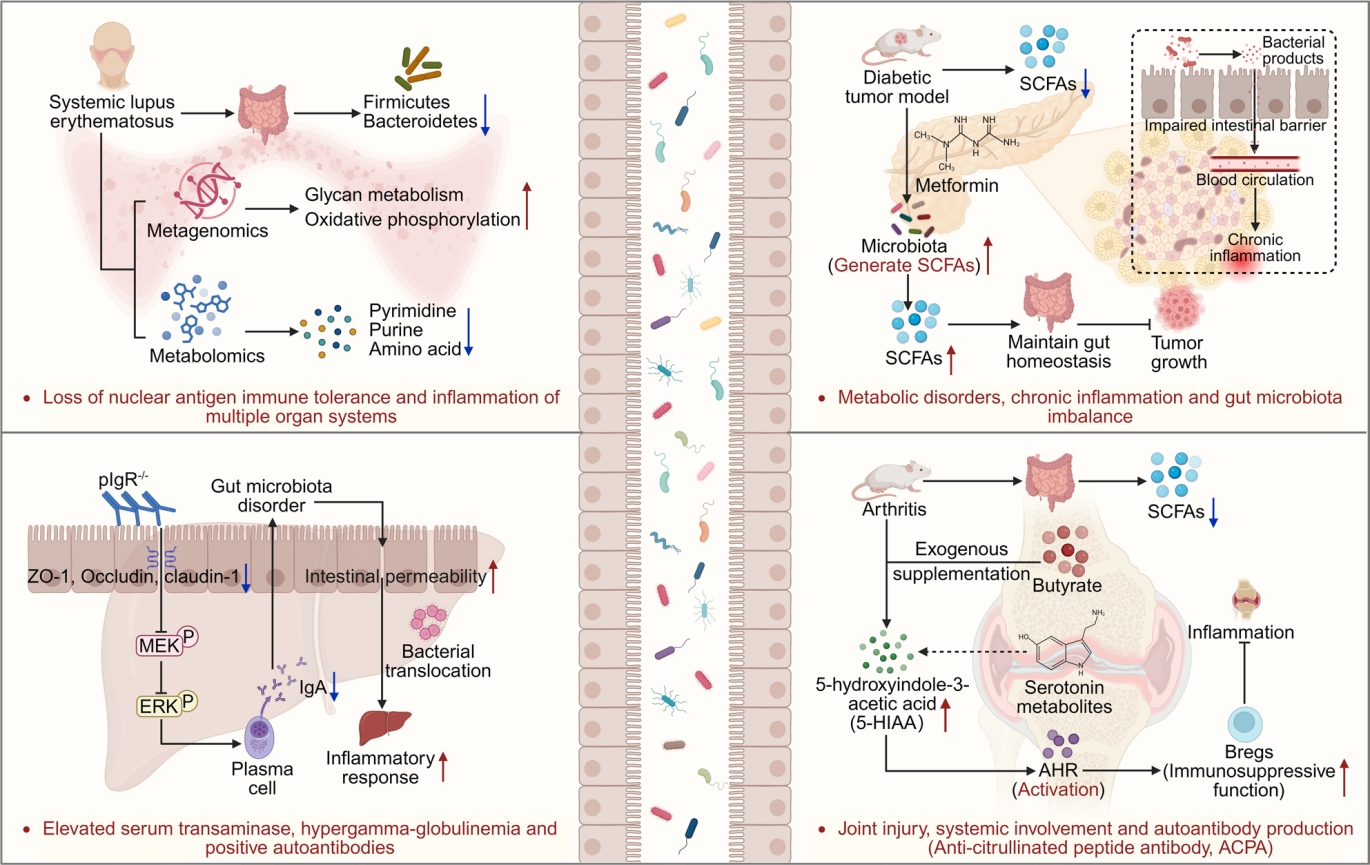
The interaction mechanism between GM and AD and the regulation of immune metabolism. The occurrence of AD, such as SLE, DM, and RA is closely associated with the composition of the GM and abnormal metabolites. In patients with SLE, genes involved in glycan metabolism genes are upregulated, while pyrimidine metabolites are decreased in the GM. Patients with DM exhibit increased gut permeability due to microbiota imbalance, leading to chronic inflammation. Additionally, distinct microbiota alterations are observed in both T1D and T2D. Metformin can modulate the microbiota by increasing the levels of SCFAs. In patients with RA, gut SCFAs levels are reduced. Exogenous supplementation with butyrate can activate the AHR thereby inhibiting joint inflammation. Furthermore, deficiency of the pIgR, a key component of the gut immune barrier, disrupts tight junctions in gut epithelium, causes microbiota dysbiosis, and exacerbates autoimmune hepatitis. These fingdings highlight the central role of GM-immune interactions in the pathogenesis and treatment of AD. Created in https://BioRender.com

ADs is chronic immune disease characterized by dysregulation of immune system, which ultimately results in a loss of self-antigen tolerance [186]. Increasing evidence indicates that the GM plays a crucial role in the onset and progression of various ADs, including multiple sclerosis (MS) [187], diabetes mellitus (DM) [188], systemic lupus erythematosus (SLE) [189], and rheumatoid arthritis (RA) [190]. Notably, changes in diet and the use of antibiotics have significantly altered the composition and diversity of GM, which may be one of the important factors contributing to the rise in AD incidence [191]. For example, SLE is a complex AD characterized by a loss of immune tolerance to nuclear antigens, which in turn triggers inflammatory responses in multiple organ systems [192]. The relative abundance of *Firmicutes* and *Bacteroidetes* in the GM of SLE patients is lower than that of healthy individuals. Metagenomic analyses revealed abnormalities in metabolism-related pathways in SLE, especially significantly upregulated expression levels associated with glycan metabolism and oxidative phosphorylation [193]. Additionally, metabolomics analysis revealed significantly reduced levels of metabolites associated with pyrimidine, purine, and amino acid metabolism in the fecal metabolites of SLE patients [191, 194] **(**Fig. 4**)**.

DM is one of the fastest growing metabolic diseases worldwide [195]. Numerous clinical studies have confirmed that patients with DM not only experience metabolic disorders and chronic inflammation but also exhibit significant GM dysregulation [188, 196]. For example, an imbalance in the Bacteroides/Firmicutes ratio is associated with increased gut permeability, allowing bacterial metabolites to enter the bloodstream through a compromised gut barrier, which triggers the chronic inflammatory response characteristic of DM [188]. It is important to note that GM dysregulation varies significant among different types of diabetes. For instance, studies have found that the relative abundances of *Bifidobacterium stercoris*, *Bacteroides intestinalis*, *Bacteroides cellulosilyticus*, and *Bacteroides fragilis* are higher in the GM of individuals with type 1 diabetes (T1D) [197]. In contrast, patients with type 2 diabetes (T2D) show enrichment of certain pathogenic bacteria, including *E. coli*, *Clostridium species*, *Bacteroides caccae*, and *Eggerthella lenta* [198]. Additionally, the content of SCFAs, such as acetic acid, propionic acid, and isobutyric acid, is significantly reduced in DM patients, whereas the levels of these are increased following Metformin intervention [199]. Metformin treatment also promotes the growth of SCFA-Producing bacteria, incliding genera such increased *Ruminococcaceae*, *Clostridiales*, *Anaerovorax*, *Odoribacter*, and *Marvinbryantia*, which have been shown to maintain gut homeostasis and inhibit tumor growth in the DM tumor models [199] **(**Fig. 4**)**.

The gut immune barrier is a crucial component in maintaining body homeostasis and comprises both the innate and adaptive immune systems [50]. Impairment of its function leads to reduced pathogen clearance and disruption of gut homeostasis, thereby increasing the risk of AD [200]. It has been found that the polymeric immunoglobulin receptor (pIgR), a key molecule in mucosal immunity, when deleted, inhibits the MEK/ERK signaling pathway, reduces the secretion of IgA, disrupts gut epithelial tight junction proteins, and cause GM imbalance [201]. This disruption of gut barrier function further increases gut permeability and facilitates bacterial translocation to the liver, exacerbating liver inflammatory responses in experimental autoimmune hepatitis (EAH) models [201]. Meanwhile, it has been confirmed that defects in thymic central immune tolerance can also alter the composition of the GM, and the disrupted microbiota may further exacerbate the progression of conditions such as autoimmune hepatitis [202]. Notably, regulatory T cells (tTregs) derived from the thymus play a dominant role in maintaining immune tolerance to the GM, rather than Treg induced locally in the gut [203]. This discovery further highlights the crucial role of thymus in shaping the T cell receptor repertoire against the GM. Beyond the gut barrier mechanism, recent studies have revealed the regulatory role of GM metabolites in RA [190]. RA is an AD characterized by joint damage, systemic involvement, and the production of autoantibodies such as anti-citrullinated peptide antibodies (ACPA) [204, 205]. Research has shown that SCFAs levels in the gut of RA patients and arthritis mice are significantly lower than those in healthy controls. Exogenous butyrate supplementation increases levels of the serotonin-derived metabolite 5-hydroxyindole-3-acetic acid (5-HIAA). enhances the immunosuppressive function of Bregs by activating aryl hydrocarbon receptors (AhR), and ultimately inhibits the pathological progression of arthritis [128]**(**Fig. 4**)**.

### Other diseases

#### Metabolic disorders

Obesity is closely associated with a reduction in GM diversity and a high incidence of micronutrient deficiencies [206]. Substantial evidence indicates that an abnormal GM composition and increased gut permeability (“leaky gut”) play key roles in the chronic inflammation commonly observed in obesity and diabetes [207]. Specifically, the diminished capacity of the microbiota associated with obesity to metabolize ethanolamine exacerbates gut permeability, triggers inflammation, and impairs glucose metabolism. However, restoring ethanolamine metabolic capacity through novel probiotic therapy can reverse these pathological changes [207]. Mechanistically, total saponins of Panax notoginseng (PNS) increase the abundance of *Akkermansia muciniphila* and *Parabacteroides distasonis*, activate the leptin-AMPK/STAT3 signaling pathway, promoting the thermogenic activity of brown adipose tissue and the formation of beige adipocytes, thereby improving diet-induced obesity by enhancing energy expenditure [208].

Furthermore, lipopolysaccharides (LPS) derived from the GM in obese individuals can activate the TLR4 pathway, inducing bone marrow macrophages (BMMs) senescence and grancalcin (GCA) secretion, thereby exacerbating bone degeneration associated with obesity [209]. In addition to the mechanisms mediated by microbial metabolites, specific bacterial communities and their metabolites also directly regulate host metabolism. For example, *Lactobacillus reuteri* ZJ617 provides substrates for bacteria that produce spermidine, promoting spermidine synthesis and thereby alleviating metabolic syndrome(MetS) [210]. Conversely, tryptamine and aniline, produced through dietary amino acid catabolism mediated by *Ruminococcus gnavus*, impair insulin sensitivity by activating the TAAR1-MAPK/ERK signaling pathway, thus promoting insulin resistance associated with GM imbalance and T2D [211]. Moreover, studies using 16 S rDNA amplicon sequencing and metabolomic profiling have demonstrated that purified citrus polymethoxyflavone-rich extract (PMFE) can effectively alleviate GM imbalance and improve MetS induced by a high-fat diet by regulating branched-chain amino acid (BCAA) metabolism [212].

#### Allergic and skin conditions

Allergic diseases are chronic immune disorders, with an increasing incidence worldwide, affecting approximately 20% of the population, particularly children [213, 214]. The skin and gut are the two primary habitats of microbiota, and microbial imbalance is considered a key environmental factor that triggers and exacerbates allergic reactions [214]. Numerous studies have demonstrated that imbalances in the composition and function of the GM can disrupt the abnormal immune responses, thereby influencing the onset and progression of various allergic diseases, including asthma and atopic dermatitis [215]. For example. recent studies has shown that the *Bifidobacterium longum* CCFM1029 can effectively reshape the GM composition, significantly increase levels of indole-3-carbaldehyde (I3C) in feces and serum, and maintain the abundance of *Lachnospiraceae*, which is associated with tryptophan metabolism in the GM [216]. Moreover, this bacterial strain can upregulate tryptophan metabolism in the gut via the “gut-skin axis”, produce I3C, and activate the AHR-mediated immune response, thereby alleviating symptoms of atopic dermatitis [216].

Moreover, the skin and GM of patients with psoriasis exhibit significant dysregulation. At the level of skin microecology, both α diversity and β diversity of the microbial community in the lesion areas of patients with psoriasis are significantly lower than those in healthy individuals [217]. Specifically, the relative abundances of *Lactobacilli*, *Burkholderia spp*., and *P. acnes* were significantly decreased, while the genera *C. simulans*,* C. kroppenstedtii*, *Finegoldia*, and *Neisseriaceae* species are significantly enriched [218]. At the level of the GM, the microbial community structure of patients with psoriasis has also undergone notable changes. Compared with the healthy controls, the relative abundances of the *Firmicutes* and *Actinobacteria phyla* in the gut of patients with psoriasis are significantly increased [219]. At the species level, the abundances of *Ruminoccocus gnavus*, *Dorea formicigenerans*, and *Collinsella aerofaciens* are significantly elevated, whereas those of *Prevotella copri* and *Parabacteroides distasonis* are significantly decreased [219].

#### Neurological and other disorders

The occurrence of autism spectrum disorders (ASD) is often accompanied by a loss of gut barrier function, abnormal microglial activation, and an imbalance of neurotransmitters [220]. Studies have shown that improving the GM can alleviate inflammatory disorders in the gastrointestinal tract, regulate neuroinflammatory mediators and neurotransmitters, and thereby mitigate the core symptoms of ASD [221]. In animal model studies, KD intervention significantly increased the relative abundance of beneficial bacteria (such as *Akkermansia* and *Blautia*) in BTBR and C57 mice, while also reversing the abnormal elevation of Lactobacillus in the feces of BTBR mice [222]. Consistent with human studies, there are significant differences in the gut microbiota β-diversity between oASD and oTD mice. Specifically, the abundance of Bacteroides and Parabacteroides is reduced in oASD mice, while the levels of *Akkermansia*, Sutterella, and *Lachnospiraceae* are increased [223]. Clinical studies have also confirmed structural deviations in the gut microbiota of ASD patients [224]. Compared to healthy controls, ASD patients show significantly elevated levels of *Proteobacteria*, *Actinobacteria*, and the genus *Sutterella* in their gut [225]. In the pediatric ASD population, the richness of microbiota decreased while the evenness increased, and the Deinococcus-Thermus significantly decreased [226].

It is worth noting that the GM not only affects the central nervous system but also plays a crucial role in cardiovascular diseases. Heart failure (HF) can lead to gut congestion and barrier dysfunction, allowing bacteria and their products to enter the circulation and exacerbate the systemic inflammatory response [227]. Furthermore, the GM metabolizes dietary choline and L-carnitine to produce trimethylamine N-oxide (TMAO), which can promote the occurrence and progression of atherosclerosis by inhibiting cholesterol metabolism, activating pro-inflammatory pathways, and inducing platelet aggregation and endothelial dysfunction [228]. Clinical studies have shown that plasma TMAO levels are positively correlated with the risk of chronic HF, suggesting that TMAO may be a new target for HF treatment of [229]. In addition to promoting atherosclerosis, elevated plasma TMAO levels may also accelerate myocardial hypertrophy, aggravate mitochondrial dysfunction, and induce remodeling of the transverse tubules (T-tubules) in cardiomyocytes [230].

In conclusion, the GM, as a central hub connecting multiple diseases systems, plays a crucial role in the occurrence and progression of various diseases such as obesity, allergies, and autism by influencing metabolism, immunity, and neural signaling pathways (Table 1). A comprehensive analysis of the microbial-host communication mechanisms not only provides a new insights into the pathological processes of these diseases but also establishes scientific foundation for developing precise intervention strategies based on microecological regulation.

**Table 1:** Summary table of disease mechanisms associated with GM dysbiosis

Disease category	Specific disease	Key microbial communities/metabolite changes	Core mechanisms/pathways	Reference
Metabolic disorders	Obesity	Decreased ethanolamine metabolism capacity	Increased gut permeability induced inflammatory energy metabolism disorders	[207]
		Increased abundances of Akkermansia muciniphila and Parabacteroides distasonis	Activation of leptin-AMPK/STAT3 signaling pathway promoting the thermogenic effect of brown adipose tissue and the formation of beige adipocytes	[208]
	Metabolism syndrome	Lactobacillus ZJ617 promotes spermine synthesis	Administration of S-adenosylmethionine promotes spermine synthesis alleviates metabolic syndrome	[210]
	Type 2 diabetes mellitus (insulin resistance)	Ruminococcus gnavus colonizes excessive production of tryptamine and aniline	Activation of the TAAR1-MAPK/ERK pathway impairs insulin sensitivity	[211]
Allergic and skin conditions	Atopic dermatitis	Restoring the composition of GM; Increases feces and serum indole-3-formaldehyde (I3C) levels; Maintaining the abundance of Lachnospiraceae	Upregulating tryptophan metabolism through the gut-skin axis; Promotes I3C generation; Activates the AhR signaling pathway; Alleviates symptoms of atopic dermatitis.	[216]
	Psoriasis (skin, gut microbiota)	Decreased abundance of Lactobacilli, Burkholderia spp. and P. acnes; Increased abundance of C.simulans, C. kroppenstedtii, Finegoldia and Neisseriaceae; Increased abundance of Firmicutes and Actinobacteria.	Modulation of skin barrier function triggers inflammatory responses; Th2-type signal transduction is involved.	[218, 219]
Neurological and other disorders	ASD	Increased the relative abundance of Akkermansia and Blautia; Decreased levels of Lactobacillus in feces (BTBR mice).	Modifying lipid peroxidation levels and superoxide dismutase activity in the BTBR brain region; Reduces oxidative stress; Reconstructing the gut-brain axis.	[222]
	ASD (Animal Model)	Decreased the abundance of Bacteroidetes, Bacteroides and Parabacteroides; Increased abundance of Akkermansia, Sutterella and Lachnospiraceae.	Improving behavioral abnormalities Regulates neuronal excitability in the brain.	[223]
	ASD (children)	Increased uniformity of microbial community composition; Deinococci and Holophagae were significantly lower; Decreased ornithine levels and elevated valine levels.	Increased ASD risk through metabolic pathway peptides/nickel transport system	[226]
	Atherosclerosis	Produce Trimethylamine N-oxide (TMAO)	Inhibiting cholesterol metabolism; Activating pro-inflammatory pathways; Inducing platelet aggregation and endothelial dysfunction; Promote the occurrence and progression of atherosclerosis	[228]
	Heart failure	Elevated plasma TMAO levels	Accelerate myocardial hypertrophy; Aggravate mitochondrial dysfunction; Induce remodeling of the transverse tubules (T-tubules) in cardiomyocytes	[230]

## Therapeutic strategies based on gut microbiota and gut immune regulation

### Probiotics, prebiotics and synbiotics intervention

**Fig. 5:**
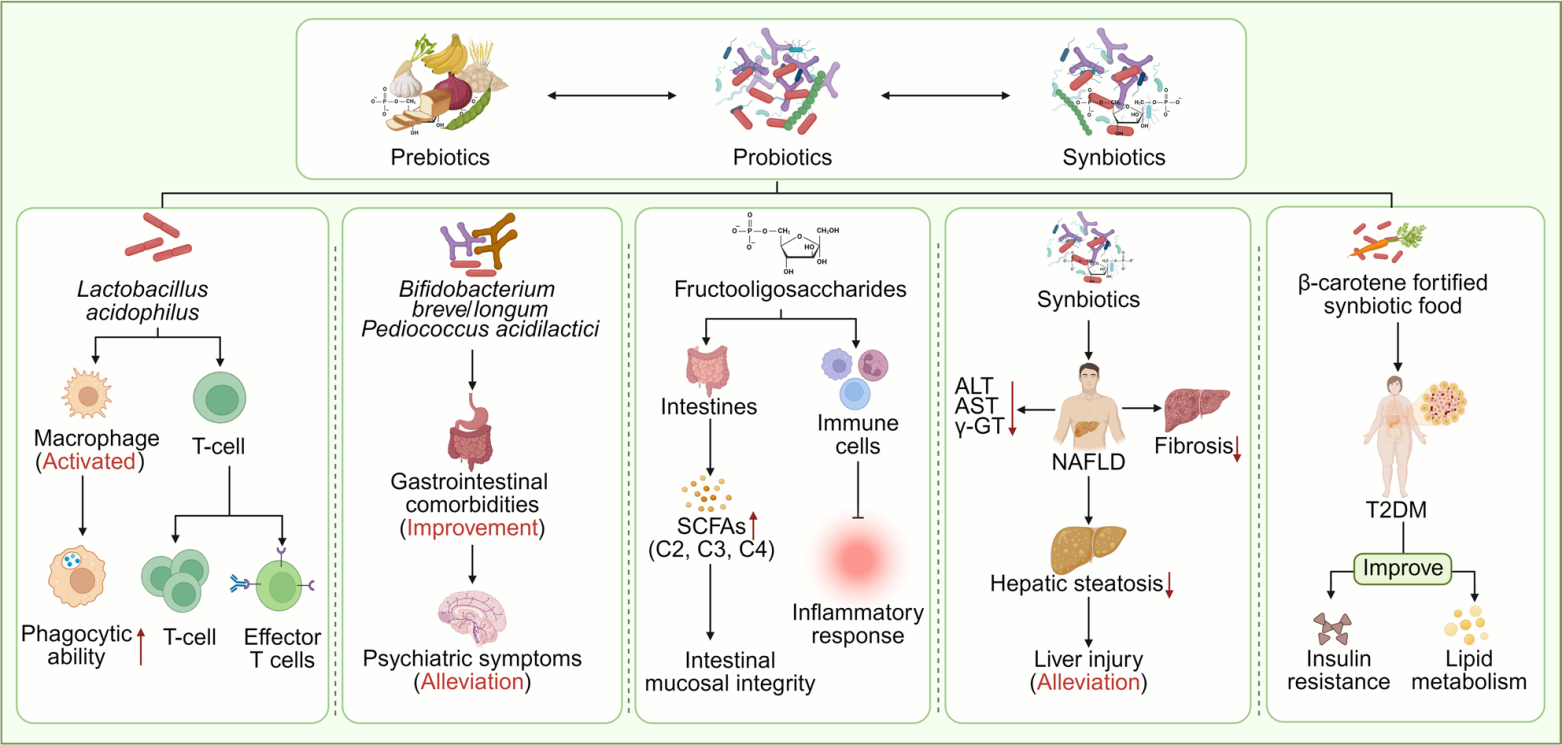
Synergistic mechanism of probiotics-prebiotics-synbiotics on GM, immune regulation and disease intervention. Probiotics (*Lactobacillus acidophilus*) can activate the immune functions of macrophages and T cells, and their combined strains can improve both mental and gastrointestinal symptoms in patients with MDD by increasing serotonin levels. Prebiotics, such as FOS and IMO, serve as nutritional substrates for beneficial bacteria like *Bifidobacterium.* These prebiotics promote the production of SCFAs, which help maintain the intestinal barrier and inhibit inflammatory responses. The combination of probiotics and prebiotics supports the colonization of beneficial bacteria, collaboratively regulating immunity, improving liver enzyme indicators in non-alcoholic fatty liver disease, and alleviating insulin resistance in type 2 diabetes. Created in https://BioRender.com

Probiotics are defined as “active microorganisms that provide health benefits to the host when administered in sufficient quantities”, including bacteria, yeasts, and other microbes. Their primarily mechanism involves the bidirectional regulation of GM homeostasis and the host immune response [231, 232]. At the microbiota regulation level, probiotics optimize the composition of the GM and enhance its diversity by competitively inhibiting the colonization and overgrowth of potential pathogens (such as *Klebsiella* and *E. coli*) in the gut mucosa [233, 234]. Typical effects include a reduction in the *Firmicutes*/*Bacteroides* ratio and the promotion of beneficial bacteria recovery, such as *Lactobacillus* species [235]. At the immunomodulatory level, probiotics, such as *Lactobacillus acidophilus*, can directly activate gut immune cells, stimulate macrophages to enhance phagocytic activity, promote T cell proliferation and differentiation, and coordinate the regulation of cellular and humoral immune responses, thereby strengthening gut immune barrier function [236–238] **(**Fig. 5**)**.

For instance, the probiotic *Lactobacillus* plantarum-based strain HNU082 (Lp082) restores the biological barrier by modulating the gut microbiome and increasing the production of SCFAs [239], Specifically, Lp082 reduces the levels of IL-1β, IL-6, TNF-α, MPO, and IFN-γ, while increasing the levels of IL-10 and TGF-β1/β2, It also inhibits the NF-κB signaling pathway and enhances the immune barrier [239]. *Bifidobacterium* not only upregulates inhibitory regulatory T cells, maintains gut barrier function, regulates the activity of DCs and macrophages, and suppresses gut Th2 and Th17 responses, but also alleviates symptoms related to leaky gut by reducing inflammation, improving gut barrier integrity, and promoting the rapid recovery of the GM [10, 240]. Additionally, multi-strain probiotic formulations can improve gut barrier integrity by regulating tight and adherens junction proteins [241]. In psychiatry and neurology, Major Depressive Disorder (MDD), a prevalent mental illness worldwide, is often comorbid with gastrointestinal dysfunction such as gut motility disorder [242, 243]. Probiotics interventions in MDD highlight the mechanisms of the “microbiota-gut-brain axis”. Combined preparation containing *Bifidobacterium oblatum* CCFM1025, *Bifidobacterium longum* CCFM687, and *Streptococcus lactis* CCFM6432 significantly alleviates both the psychiatric symptoms and the associated gastrointestinal dysfunction in MDD patients [243]. In chronic stress models, this preparation also improves gut peristalsis, with effects closely linked to the regulation of serotonin (5-HT) levels [243, 244] **(**Fig. 5**)**.

Prebiotics are defined as “substrates selectively utilized by host microorganisms to confer health benefits” [245]. They are commonly found in plant-based foods such as onions, asparagus, garlic, and wheat, providing specific nutrient substrates for beneficial bacteria (such as *Bifidobacterium* and *Lactobacillus*) and promoting their growth and metabolism. This, in turn, indirectly optimizes the gut microecology [246–248]. Typical prebiotics include fructooligosaccharides (FOS), isomaltooligosaccharides (IMO), and xylo-oligosaccharides (XOS) [247]. For example, Fos can promote the production of SCFAs such as butyric acid, propionic acid, and acetic acid in the gut. These metabolites not only provide energy for gut epithelial cells and maintain the integrity of the gut mucosal barrier but also regulate immune cell activity and inhibit inflammatory responses [94, 249]**(**Fig. 5**)**. Studies have confirmed that FOS and GOS regulate adipogenesis signaling pathways (SREBP-1c, ACC, and FAS) and fat decomposition (ATGL), while reducing inflammatory markers such as p-NFκB-65, IL-6, iNOS, COX-2, TNF-α, IL-1β, and nitrotyrosine [250]. Additionally,, they significantly increase the abundance of beneficial gut bacteria such as *Bacteroides acidifaciens* and *Bacteroides dorei* [250]. In mice with HFHC and MCD diets, FOS can reshape the GM structure by significantly increasing the abundance of *Bacteroidetes*, *Klebsiella*, and *Clostridium perfringens*, while reducing the abundance of Wart microorganism (at the phylum level) and Fissicatena groups (at the genus level) [251]. Human milk oligosaccharides (HMOs) also possess prebiotic properties, they not only promote the growth of beneficial bacteria but also prevent pathogens from binding to epithelial cells. Microorganisms such as *Bifidobacterium* and *Lactobacillus*, along with and HMOs transmitted to infants through breast milk, jointly promote the proliferation and colonization of GM [252, 253]. It is important to note that prebiotic interventions must consider individual host differences. In patients with IBS or IBD, prebiotics may exacerbate symptoms by accelerating gut peristalsis and fermentation processes [254, 255]. This undersocres the need for future research needs to integrate the host’s genetic background, baseline microbiota characteristics, and disease phenotypes to develop precise prebiotic intervention strategies that optimize clinical translation.

Synbiotics are defined as “mixtures composed of substrates selectively utilized by live microorganisms and host microorganisms, which confer health benefits on the host”, to more effectively regulate the GM-host immune interaction network through the synergistic combination of probiotics and prebiotics [256, 257]. The underlying mechanism is that prebiotics provide exclusive nutritional substrates for co-consumed probiotics, enhancing their colonization, survival, and metabolic activities within the complex gut environment. Simultaneously, prebiotics stimulate the growth of beneficial indigenous bacteria. Together, these effects amplify the beneficial regulation of the host immune system, such as enhanced immune defense and the promotion of an anti-inflammatory environment [258]. Research evidence indicates that supplemention with probiotics, prebiotics, or synbiotics can significantly improve liver enzyme profiles-sincluding alanine aminotransferase, aspartate aminotransferase, and γ-glutamyl transferase-as well as reduce liver fibrosis and the degree of steatosis in patients with Non-Alcoholic Fatty Liver Disease (NAFLD), thereby mitigating liver injury [259]. A randomized controlled trial (RCT) involving 102 participants demonstrated that after 6 weeks of supplementation with a synbiotic preparation containing specific lactic acid bacteria and beta-carotene (along with inulin prebiotics), insulin resistance (IR) and lipid metabolism indicators in patients with T2DM were significantly improved [260] **(**Fig. 5**)**.

In conclusion, probiotics, prebiotics, and synbiotics key intervention strategies targeting gut microecology, profoundly influence both gut and systemic immune homeostasis by reshaping the structure of the GM and regulating the production of metabolic products.

### Fecal microbiota transplantation

**Fig. 6:**
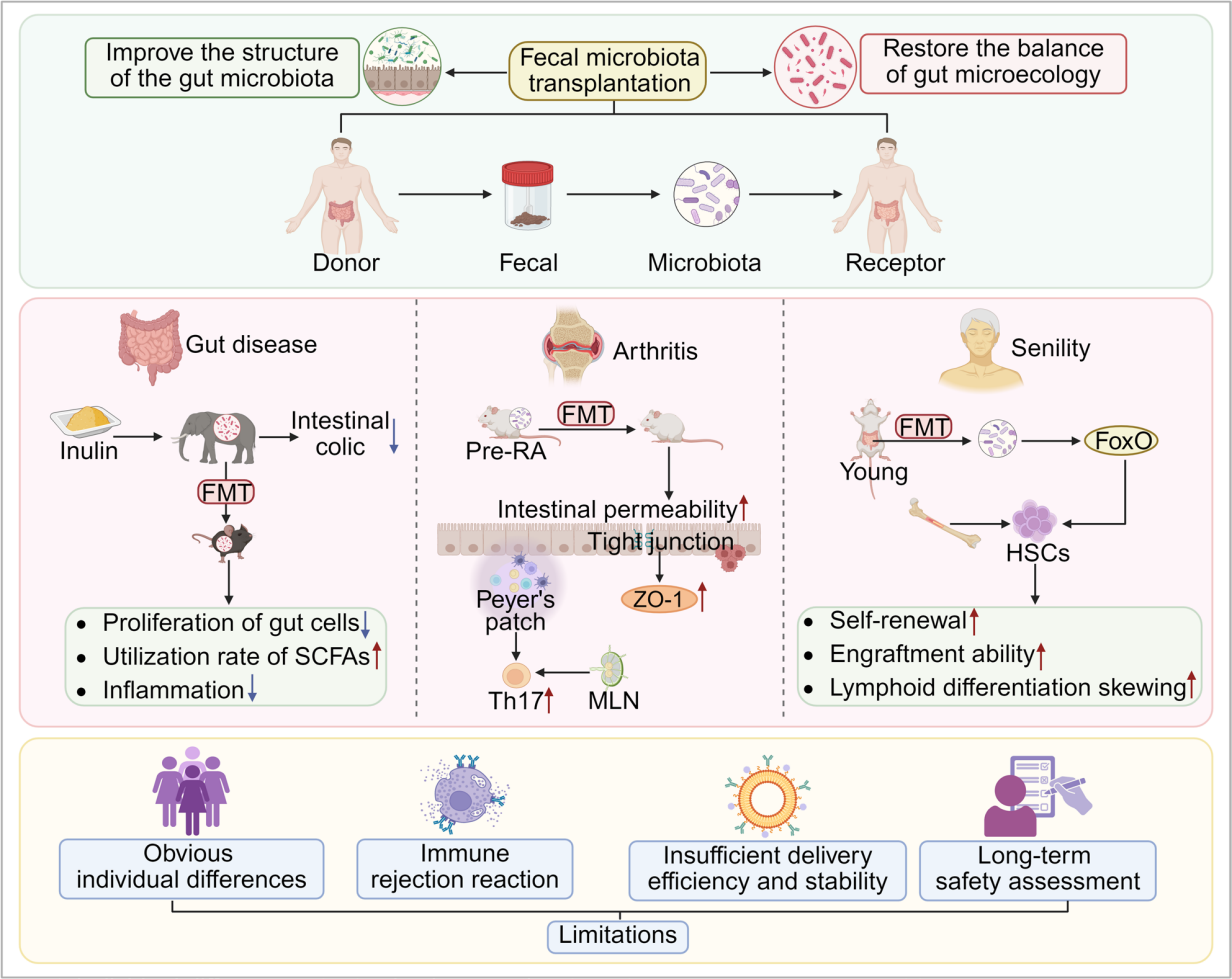
The therapeutic mechanism and existing challenges of FMT. FMT reconstructs the gut microecology, regulates the microbiota composition, and activates immune-inflammatory pathways. Inulin intervention repairs the gut barrier by promoting cell proliferation and enchancing the utilization rate of SCFAs. Studies in RA mouse models have shown that FMT influences immunopathology by increasing gut permeability, upregulating ZO-1 expression, and promoting the differentiation of Th17 cells in the MLNs. In aging modes, FMT from young mice increases the abundance of *Lachnospiraceae*, modulates tryptophan metabolism, activates FoxO signaling, and reverses HSCs aging. FMT hold significant therapeutic potential for gut-related, autoimmune, and aging-associated diseases. However, challenges such as individual variability in response, immune rejection, donor stability, and long-term safety necessitate the development of standardized protocols. Created in https://BioRender.com

Fecal Microbiota Transplantation (FMT) is a therapeutic strategy that reconstructs the gut microecosystem and modulates microbiota-immune interactions by transplanting functional microbiota from the feces of healthy donors into the patient’s gut [261] **(**Fig. 6**)**. The core mechanism involves in the ability of healthy donor microbiota to colonize and proliferate within the recipient’s gut, thereby correcting microbial dysbiosis and restoring the balance of the gut microecological environment [262–264]. Additionally, the newly colonized microbiota enhances gut immune defense and suppresses inflammatory responses through pathways such as activating immune cells and regulating cytokine secretion [265, 266]. Notably, FMT-mediated microbiota remodeling can synergistically enhance the host’s anti-tumor immune response, thereby improving the clinical efficacy of tumor immunotherapy regimens, including immune checkpoint inhibitors (ICIs) such as PD-1/PD-L1 antibodies [267]. In patients with SLE, FMT significantly enriches microbiota that produces SCFAs, reduces inflammation-associated bacterial genera, and is accompanied by increased SCFAs levels and decreased proportion of IL-6 and CD4^+^ memory/naive T cells in peripheral blood [189]. In colitis models, FMT significantly reduces levels of inflammatory markers myeloperoxidase (MPO) and eosinophil peroxidase (EPO), as well as various pro-inflammatory factors, whil increasing IL-10 secretion [268].

At present, FMT demonstrates significant potential in the treating various human diseases. However, its clinical application has not yet gained widespread acceptance. Oral microbiome capsules, as an innovative delivery method, offer the potential to enhance patient compliance [269]. Compared with traditional fecal transplantation, oral fecal intragastric administration (OFG) is enriched with *Lachnospiraceae* and butyrate. Butyrate alleviates ferroptosis in acute liver injury (AILI) by activating the AMPK-ULK1-p62 signaling axis and induces mitochondrial autophagy and Nrf2 antioxidant responses [269]. Clinical studies have shown that, regardless of metformin use, FMT can significantly improve insulin resistance, body mass index, and GM abundance in patients with T2DM by colonizing donor-derived microbiota [270]. When combined with prebiotics (a 9:1 ratio of galactose to fructooligosaccharides), FMT can repair the gut barrier, promote nutrient absorption, relieve the symptoms of UC, and simultaneously increase the abundance of *Lactobacillus*, *Bifidobacterium*, and SCFAs [271]. In an atopic dermatitis (AD) model, FMT significantly improved the GM composition in mice by increasing the *Firmicutes*/*Bacteroidetes* ratio and enhancing the abundance of butyrate-producing bacteria (e.g., *Erysipelothricaceae*, *Eubacteriaceae*) [272].

Cross-species research further highlights the extnesive role of FMT. For example, inulin intervention can cure colic in sick elephants and improve the composition of their GM. Transplanting their feces into mice promotes the proliferation of gut epithelial cells, increases the utilization rate of SCFAs, maintains gut barrier function, and reduces inflammatory responses [273] **(**Fig. 6**)**. Beyond gut diseases, FMT has gaenered significant attention in autoimmune diseases. In studies of rheumatoid arthritis (RA), microbiota transplantation from patients in the pre-RA stage increased gut permeability in recipient mice, upregulated the expression of the tight junction protein ZO-1, and promoted the expansion of Th17 cells in MLN and Peyer’s patches [274] **(**Fig. 6**)**. A breakthrough in aging research demonstrated that FMT from young mice significantly enhances the hematopoietic reconstitution capacity of senescent hematopoietic stem cells (HSCs) by reshaping the GM composition in elderly recipients-such as increasing the abundance of *Trichospiraceae*-and modulating the metabolite profile, including tryptophan metabolism-related products. This process activates the FoxO signaling pathway, promotes lymphoid differentiation, reverses bone marrow output bias, and ultimately reduces the risk of hematological diseases [275] **(**Fig. 6**)**.

The mechanism of FMT extends beyond the simple concept of bacterial transfer. Current evidence indicates that its efficacy arises not only from the colonization and reconfiguration of donor bacteria in the recipient’s gut but also involves the collaborative participation of various non-bacterial components. At the level of bacteriophages, FMT can induce a significant shift in the recipient’s gut phage community toward that of the donor, altering the community composition while enhancing both α diversity and temporal variability [276]. Clinical studies have confirmed that phages from healthy donors can temporarily reshape the recipient’s GM and improve glucose fluctuations in patients with metabolic syndrome [277]. Mechanistically, bacteriophages induce the production of IFN-γ via the TLR9 pathway, thereby modulating gut immunity. In germ-free mice, bacteriophage transplantation promotes immune cell proliferation, whereas increased bacteriophage levels exacerbate colitis through the TLR9/IFN-γ axis. Notably, bacteriophages derived from patients with active UC exhibit a stronger capacity to induce IFN-γ than those from healthy controls, suggesting that bacteriophages can influence host health by regulating mucosal immunity [278]. Furthermore, FMT from dyslipidemic donors (FMT-dd) can increase the abundance of fecal *Bacillus* and *Ruminococcaceae* UCG-010-menbers of the rumen bacterium family-thereby reshaping the GM of mice. This promotes elevated serum levels of cholic acid (CA), deoxycholic acid (CDCA), and lithocholic acid (DCA), subsequently inhibiting bile acid synthesis and activating the hepatic FXR-SHP axis, which induces mild lipid metabolism disorders [279]. This further suggests that, compared to hepatic FXR, gut FXR may play a more critcial role in regulating lipid metabolism via microbial community-bile acid interactions, particularly in the pathological context of diet-induced dyslipidemia.

In summary, the therapeutic effects of FMT arise from the synergistic actions of multiple components and pathways. These include direct immune regulation by non-bacterial elements such as bacteriophages, ecological remodeling driven by bacterial reconfiguration, and modulation of host signaling pathways through the overall metabolic function of the microbiota. Nevertheless, FMT faces several significant limitations that require urgent attention [280]. Biological varibility among individuals-such as chronic inflammatory environments in CRC patients that inhibit GM colonization-and severe immune rejection reactions pose challenges [281, 282]. Technically, FMT is constrained by insufficient standardization of donor screening, varibility in the efficiency and stability of delivery methods (e.g., oral microbiota capsules (OMC) and colonoscopy), and a lack of long-term safety date [283–285]**(**Fig. 6**)**. Future efforts should focus on optimizing donor screening criteria, developing precise delivery systems, and thoroughly elucidating the mechanisms underlying microbiota-host immune interactions to advance FMT into a personalized, safe, and effective therapeutic strategy.

### Combined application of immunomodulatory drugs and microbiome targeting therapy

The integrated application of immunomodulatory drugs and microbiota-targeted therapies represents an emerging therapeutic strategy that offers dual advantages in disease treatment. This approach synergistically combines the direct regulatory effects of immunomodulatory drugs on the immune system with the restorative effects of microbiota-targeted therapies on the gut microecology [286, 287]. However, the precise mechanisms by which the GM modulates immune responses and influences the efficacy of immunotherapy have yet remain to be fully elucidated.

**Fig. 7:**
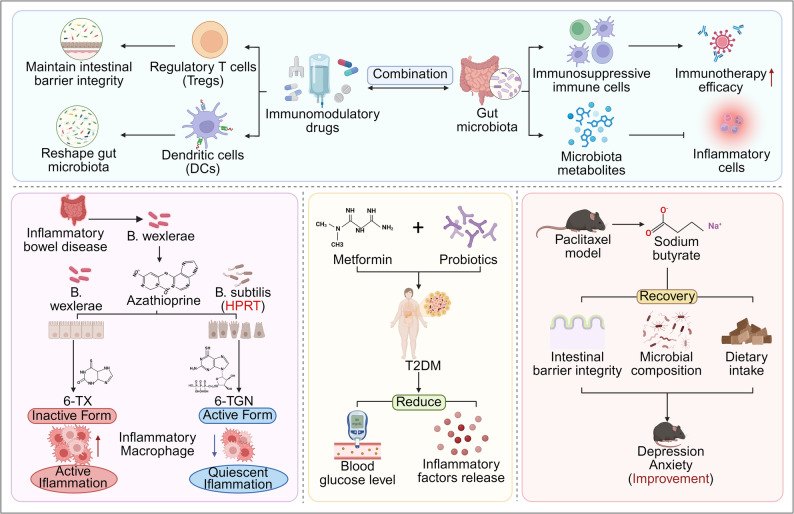
Integration mechanism and synergistic therapeutic potential of immunomodulatory drugs and microbiota targeted therapy. The integrated strategy combining immunomodulatory drugs and microbiota-targeted therapy regulates immune-related diseases through dual mechanisms. Immunomodulatory drugs directly modulate the immune system, such as enhancing the function of Tregs and reshaping antigen presentation by DCs. In contrast, microbiota-targeted therspies retore gut microecology by promoting the production of metabolites such as SCFAs and tryptophan derivatives, which inhibit inflammation. Long-term use of immunotherapy drugs may cause dysbiosis, whereas microbiota-targeted therapies, including probiotics and FMT) can restore microbial balance and enhance the therapeutic efficacy. *B. subtilis* can rescue azathioprine treatment failure in IBD by increasing 6-TIMP, and metformin combined with probiotics improves blood glucose control in T2D. Additionally, sodium butyrate restores paclitaxel-induced gut barrier injury and alleviates its neurological side effects, highlighting the bidirectional regulatory relationship underlying the synergistic of therapeutic potential and mechaisms between these approaches. Created in https://BioRender.com

The core of this joint strategy is a bidirectional interaction and regulatory mechanism. On one hand, immunomodulatory drugs can not only directly enhance the function of Tregs and maintain the integrity of the gut barrier [288], but also indirectly reshape the composition and diversity of the GM by modulating the antigen-presenting capacity of DCs [289]. On the other hand, the GM plays a crucial role in maintaining both local and systemic immune homeostasis. Specific symbiotic bacteria, such as *Bacteroides fragilis*, or their metabolites (SCFAs, Trp metabolites, and BAs) can promote the function of immunosuppressive cells, suppress inflammatory responses, and thereby enhance the efficacy of immunotherapy [290–292] **(**Fig. 7**)**. Mechanistic studies have demonstrated that *Bacteroides fragilis* (*B. fragilis*) promotes the maturation of intratumoral DCs and stimulates Th1 responses in tumor-drainage lymph nodes by activating TLR2/TLR4 recognition of microbe-associated molecular patterns (MAMPs), thereby enhancing the efficacy of ICI [293]. Colonization by *B. fragilis* can also correct abnormal CD4^+^ T cell populations and the Th1/Th2 imbalance observed in GF mice [294]. Similarly, oral administration of *Akkermansia muciniphila* and *E. hirae* increases the prevalence of central memory CD4^+^ T cells in the tumor microenvirment, MLN, and draining lymph nodes, while inducing DCs to produce IL-12, thereby contributing to the response to ICIs [295].

Microbiota-derived metabolites exert complex regulatory effects on immunotherapy. In pancreatic ductal adenocarcinoma (PDAC), tryptophan metabolites produced by the microbiota can activate the AhR in TAMs [296]. The absence of AhR not only inhibits tumor growth but also significantly increases the number of IFNγ^+^ CD8^+^ T cells, thereby enhancing the efficacy of ICI [296]. Interventions such as prebiotics can promote the accumulation of valeric acid, a metabolite of tryptophan degradation, reduce the kyurine/tryptophan (Kyn/Trp) ratio, inhibit Treg cells, activate effector T cells, and ultimately improve the response to anti-PD-1 therapy [297]. Notably, elevated butyrate levels can inhibit the maturation of CTLA-4 blockers (e.g., ipilimumab) and supress the CD28 signaling pathway, thereby diminishing their antitumor effects [298]. Additionally, dysbiosis can also impair the antitumor immune response by inhibiting the cGAS-STING-IFN-I signaling pathway, suppressing antigen presentation, and reduce effector T cell function [299]. Recent evidence suggests that the microbiota plays a significant role in modulating the response to ICI by regulating the immune system, particularly the abundance and function of NK cells, CTLs, and Tregs [300]. Specifically, when combined with ICIs, *P. distasonis* can significantly delay bladder cancer progression, increase intratumoral infiltration of CD4^+^ T and CD8^+^T cells, and upregulate pathways associated with the antitumor immune response [301].

Long-term immunomodulatory drug therapy may cause GM imbalance, which can affect durg efficacy or lead to adverse reactions. Microbiota-targeted interventions, such as probiotics and FMT, can effectively address the limitations of drug treatment by restoring GM balance, enhancing gut barrier function, and regulating immune responses [292, 302, 303]. In the treatment of IBD, the immunosuppressant azathioprine (AZA) suppress overactive immune response but may also disrupt GM balance. Moreover, colonization of *B. wexlerae* in IBD patients increases inflammatory macrophages and impairs the therapeutic effect of AZA in mice with enteritis [304]. Interestingly, supplementation with *Bacillus subtilis* significantly increased levels of the active metabolite 6-TIMP and successfully reversed the failure of AZA treatment induced by *B. Wexlerae*-induced in vivo [304]**(**Fig. 7**)**. In metabolic diseases, metformin combined with probiotics significantly improves blood glucose control in patients with T2DM and reduces inflammatory factors levels [199]. Paclitaxel (PTX), a chemotherapy drug fused to treat various cancers, has several limitations [305, 306]. In mouse models, PTX-induced gut barrier damage, microbiota dysbiosis,, and behaviors abnormalities such as depression and anxiety can be effectively restored by intervention with sodium butyrate (BuNa) [307] **(**Fig. 7**)**. These studies suggest that the combining microbiota-targeted therapies with immunomodulatory drugs hold potential for synergistic enhancement.

However, current clinical immunosuppressive therapies often face limitations such as inconsitnet efficacy, high cost, and serious side effects. The key challenge lies in the understanding the anti-tumor immune response mechanisms within the tumor microenvirment through the remote regulation of the GM-for example, via microbiota translocation, immune cell migration, or metabolite diffusion, Specifically, what are the exact mechanisms by which the tumor-associated microbiota recognizes the host and microbial signals, and how does it regulate the targeted migration of bacteria and immune cells to the tumor? A comprehensive analysis of the interaction mechanisms between the GM and the immune system will provide a theoretical foundation fo developing regenerative, patient-centered, low-toxicity, and cost-effective microbiota-immune combination therapies, ultimatelyenaling precise therapeutic interventions.

## Conclusion and prospect

Numerous studies have demonstrated that the relationship between GM and gut immune system is not a simple one-way interaction but rather a precise “immune-microbiota interaction axis”. The microbiota and its metabolites regulate the differentiation and function of immune cells through PRRs. Simulataneously, the gut immune system, acting as an “ecological manager”, shapes the composition and spatial distribution of the microbiota through multiple mechanisms, including SIgA, antimicrobial peptides, and mucus barriers. This bidirectonal interaction jointly maintains gut microecological homeostasis and overall organism health.

**Fig. 8:**
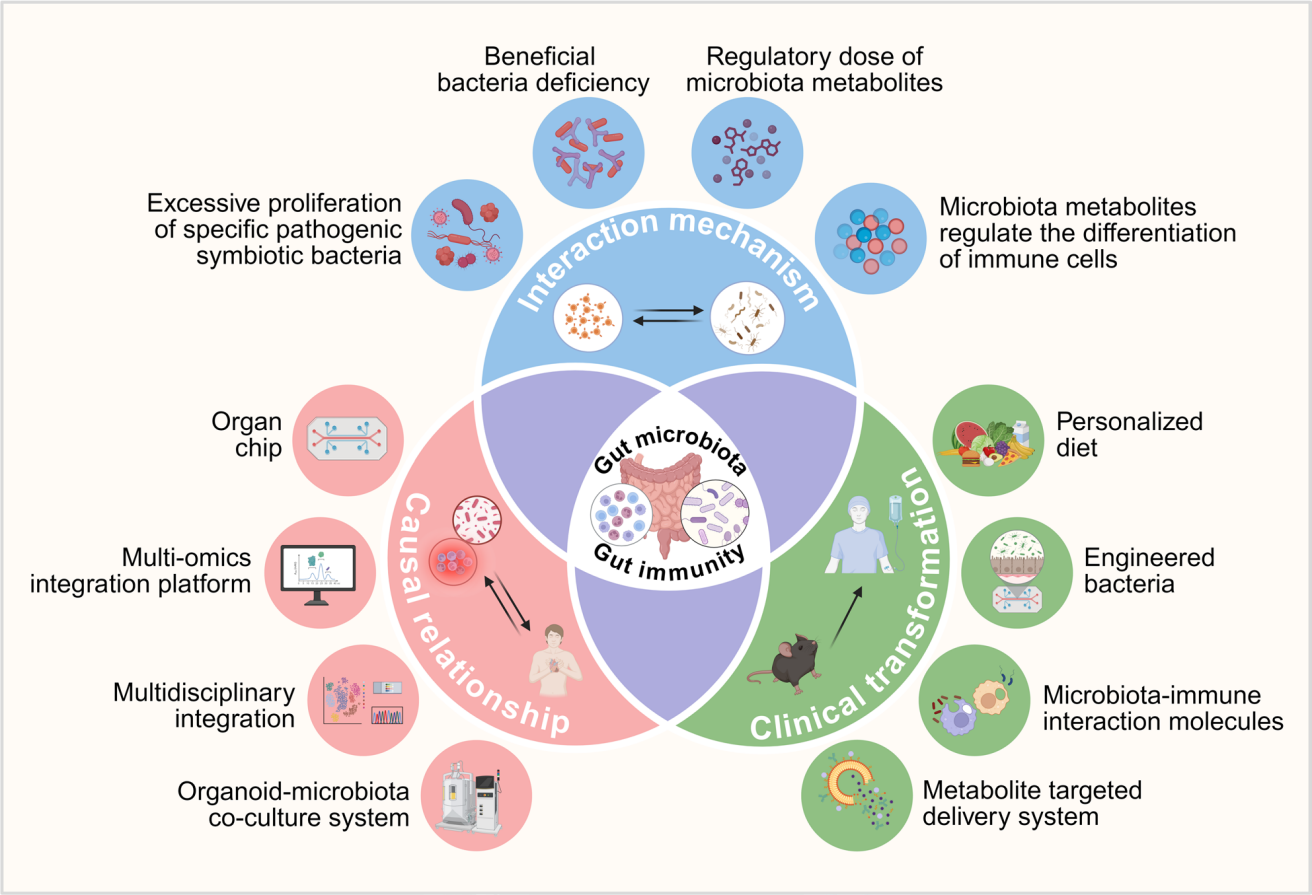
Research Challenges and Clinical Transformation Pathways of the interaction mechanism between GM and immune system. The study of GM and immune system interactions remains largely focused on the overall structure of the microbiota. The specific relationships between specific functional bacterial strains, their metabolites (such as SCFAs and Try derivatives), and immune cells have yet to be filly elucidated. Future research should integrate multi-omics approches, in vivo imaging and germ-free animal models to analyze the signaling pathways and the dose-response relationships of metabolites involved in the activation of abnormal immunity by pathogenic symbiotic bacteria, as well as the immune tolerance dificiencies caused by the absence of beneficial bacteria. There is an urgent need to clarify the causal links between microbiota imbalance and immune dysregulation using organoids-microbiota co-culture systems, organ-on-a-chip technologies, and machine learning combined with multi-omics platforms to overcome traditional limitations. Ultimately, it is essential to design engineered bacterial microcapsules, development metabolite-targeted delivery systems, and identify molecular markers to improve the pricision of individualized interventions and the stability of metabolites, thereby facilitating the translation of basic research to clinical applications. Created in https://BioRender.com

Although significant progress has been made in related research, most current studies focus primarily on the positive pathway of “dysbiosis driving immune disorder”, while the reverse regulatory mechanism-how immune abnormalities reciprocall alter and reshape the microbiota ecosystem-remains relatively underexplored. Notably, although existing studies have outlined the multi-level network through which the immune system regulates the microbiota, the dynamic evolution of this interaction during disease progression, as well as its interplay with other pathological factors, remains poorly understood. This gap in knowledge limits the clinical translation of microbiota-based intervention strategies. Therefore, before effectively integrating the GM into the disease prevention, diagnosis, and treatment systems, it is urgent to clarify the following key scientific questions:

Firstly, it is essential to conduct a precise analysis of the specific regulatory mechanisms underlying the interactions between the GM and the immune system. By integrating multiple omics technologies (e.g., metagenomics, metabolomics, spatial transcriptomics) with in vivo imaging techniques, we systematically investigated the spatial distribution characteristics and dynamic interaction interfaces of GM and mucosal immune cells within the gut barrier. Furthermore, through the analysis of host temporal samples, we elucidated in detail the regulatory mechanisms at key time points during the processes of microbiota colonization, host development, and immune maturation. For instance, we explored how specific bacterial metabolites (e.g., SCFAs, tryptophan derivatives) regulate immune cell differentiation via epigenetic modifications or metabolic reprogramming. In prospective cohorts and disease animal models, fecal transplantation, sterile animal colonization, and gene-editing techniques were used to establish causal relationships within the microbiota-immune-disease axis, focusing on the following signaling pathways: (1) the excessive proliferation of specific pathogenic symbiotic bacteria (such as *Enterococcus* and *Proteobacteria*) activating abnormal immune responses; (2) molecular compensation mechanisms of immune tolerance deficiency caused by the loss of beneficial bacteria (such as *Bifidobacteria* and *Akkermansia Muciniphila* [308]); and (3) bacterial metabolites (such as secondary bile acids and polysaccharide A) acting as signaling molecules to regulate the dose-response relationship of immune cell balance **(**Fig. 8**)**.

Secondly, it is critically important to elucidate the causal relationship between microbiota imbalance and immune dysregulation in the disease process. Current research indicates that disease onset is frequently associated with substantial alterations in microbiota composition and immune system abnormalities. However, it remains uncertain whether changes in the microbiota serve as a causative factor or a consequence of the disease. In recent years, studies have reported a close relationship between GM, gut immune-related indicators, and diseases, but significant gaps remains in the existing research framework. For example, there is a lack of a marker systems for early disease prediction and diagnosis with high specificity and sensitivity. Traditional research models, such as two-dimensional cell cultures, struggle to accurately simulate the three-dimensional structure, anoxic gradient, and metabolic characteristics of the gut microenvironment. Additionally, current analytical techniques lack the capability to rapidly, efficiently, and precisely integrate multi-omics data for a comprehensive analysis of the regulatory network among the microbiota, immune system, and host. Therefore, future research should focus on the following aspects: First, develop more reliable basic-to-clinical translational models, such as organoid-microbiota co-culture systems, organ-on-a-chip technologies and humanized mice. Second, build a machine learning-driven multi-omics integration platform (e.g., Google Cloud Life Sciences, Autoencoder) combined with surface plasma resonance(SPR) technology to deeply excavate research data, screen the key hub targets of microbiota-immune interactions, and establish a dynamic regulatory network model. Third, through the interdisciplinary intergration of bioinformatics, statistics, and computer science, achieve breakthroughs in clinical translation by analyzing the mechanisms of microbiota-immunity interactions **(**Fig. 8**)**.

Finally, significant challenges remain in efficiently translating basic research on the GM and immunity into clinical applications. Notable individual differences in GM composition and host immune response result in imprecise intervention using probiotics, prebiotics, microbiota transplantation, and other therapies. It is important to note that microbiota metabolites-such as butyric acid, bile acids, and tryptophan derivatives-are rapidly absorbed and degraded in the gut. High doses of these metabolites may trigger inflammatory responses, compromising gut stability and targeting accuracy. Therefore, there is an urgent need to develop novel intervention strategies and engineer bacteria with multifunctional, capabilities, high efficiency, and strong adaptability (e.g., butyric acid-synthesizing bacterial microcapsules) or metabolite-targeted delivery systems (e.g., hydrogels, nanoemulsions, cell carriers) to achieve precise, localized regulation of microbiota metabolites within the intestinal environment. Additionally, specific diagnostic markers for diseases caused by microbial dysregulation (e.g., IBD, CRC, and AD) are still lacking, it is essential to identify and develop key molecules involved in bacteria-immune interactions (such as specific metabolites or genes) as biomarker combinations for novel disease diagnosis, thereby improving overall therapeutic outcomes [309]. Concurrently, personalized dietary and lifestyle intervention plans should be disigned based on the GM and immune profiles individuals to enhance the effectiveness of disease prevention and treatment **(**Fig. 8**)**.

In summary, with the continuous advancement of future research techniques, we expect to more fully elucidate the molecular mechanisms underlying the interaction between the GM and immunity, as well as the multidimensional regulatory networks involved, within the framework of spatiotemporal dynamics and host genetic environment interactions. By integrating cutting-edge disciplines such as microbiome research, immunology, synthetic biology, and artificial intelligence, we can efficiently translate basic research into clinical application, thereby further promoting the paradigm shift in modern medicine from “treatment” to “health maintenance”.

## Data Availability

No datasets were generated or analysed during the current study.
